# Farnesyl phenolic enantiomers as natural MTH1 inhibitors from *Ganoderma sinense*

**DOI:** 10.18632/oncotarget.21430

**Published:** 2017-09-30

**Authors:** Ya Gao, Lihan Zhu, Jing Guo, Ting Yuan, Liqing Wang, Hua Li, Lixia Chen

**Affiliations:** ^1^ Wuya College of Innovation, School of Traditional Chinese Materia Medica, Key Laboratory of Structure-Based Drug Design & Discovery, Ministry of Education, Shenyang Pharmaceutical University, Shenyang 110016, People's Republic of China; ^2^ Hubei Key Laboratory of Natural Medicinal Chemistry and Resource Evaluation, School of Pharmacy, Tongji Medical College, Huazhong University of Science and Technology, Wuhan 430030, People's Republic of China

**Keywords:** MTH1, natural inhibitors, farnesyl phenolic compounds, anti-tumor, Ganoderma sinense

## Abstract

Cancer cells are more addictive to MTH1 than normal cells because of their dysfunctional redox regulations. MTH1 plays an important role to maintain tumor cell survival, while it is not indispensable for the growth of normal cells. Farnesyl phenols having a coumaroyl substitution are rather uncommon in nature. Eight farnesyl phenolic compounds with such substituent moiety (1–8), including six new ones, ganosinensols E–J (1–6) were isolated from the 95% EtOH extract of the fruiting bodies of *Ganoderma sinense*. Four pairs of enantiomers 1/2, 3/4, 5/6 and 7/8 were resolved by HPLC using a Daicel Chiralpak IE column. Their structures were elucidated from extensive spectroscopic analyses and comparison with literature data. The absolute configurations of C-1′ in 1–6 were assigned by ECD spectra. These compounds were predicted to have high binding affinity to MTH1 through virtual ligand screening. The enzyme inhibition experiments and cell-based assays confirmed their inhibitory effects on MTH1. Furthermore, siRNA knockdown experiments and the cellular thermal shift assay (CETSA) confirmed that the farnesyl phenolic enantiomers specifically bound with MTH1 in intact cells. Meanwhile, the low cytotoxicity of 1–8 on normal human cells further verified their good selectivity and specificity to MTH1. These active structures are expected to be potential anti-cancer lead compounds.

## INTRODUCTION

A reason for increase risks of cancer, including its aetiology, progression and metastasis, is an imbalance between the production of reactive oxygen species (ROS) and cellular anti-oxidative defenses [1]. Meanwhile, an increase ROS tension can cause damage to mitochondrial deoxynucleoside triphosphate (dNTP) pool, and it results in DNA damage [2, 3]. MTH1, a homologue of bacterial mutT, is a nucleotide pool sanitizing enzyme which converts those oxidative nucleotides such as 8-oxo-dGTP or 2-OH-dATP into their corresponding monophosphates 8-oxod-GMP or 2-OH-dAMP, respectively [2, 4]. This hydrolysis reaction ensures that these oxidized nucleotides are unable to be recognized by DNA polymerase, thus avoiding them to be incorporated into DNA, and finally preventing the mispairing of bases during replication and transversion mutations [5, 6]. It is reported that MTH1 plays an important role to maintain tumor cell survival, [7] on the contrary, normal cells do not need MTH1 [2]. Therefore, MTH1 may be only associated with tumor cell growth, which represents a new attractive therapeutic targets for the treatment of cancers these days [8]. Although there are a few chemically synthesized small molecule MTH1 inhibitors available in recent years, such as TH287/TH588, (*S*)-crizotinib, SCH51344, organometallic complexes and 8-halogenated 7-deaza-2′-deoxyguanosine triphosphates [2, 8, 9, 10]. Nevertheless, there are no any natural MTH1 inhibitors so far and the MTH1 inhibitors mentioned above are far more from clinical use. Natural products have been acknowledged as an important source for anticancer drug discovery and development [11]. Our group is making efforts to find effective MTH1 inhibitors from natural products by virtual ligand screening techniques. About 500 compounds from a small in-house database of natural products were screened against the MTH1 model *in silico* based on its X-ray structure (PDB code: 4C9X) [8] by ICM-Pro 3.8.1 molecular docking software (Molsoft, LLC) [12].

*Ganoderma sinense* (Chinese name: Lingzhi) has been used as a folk medicine for thousands of years in China and is a well-known traditional Chinese medicine. The fungal family *Ganoderma* contains more than 100 species, with wide distribution in China. *Ganoderma sinense*, together with *Ganoderma lucidum*, are recorded in Chinese pharmacopoeia for the treatment of asthma and hypertension [13]. Recent studies showed that mushrooms of *Ganoderma* possess antitumor, [14–16] anti-inflammatory, [17, 18] immune regulation, [19, 20] hepatoprotective [21] and other pharmacological effects. Previous phytochemical investigations have resulted in the isolation of more than 300 compounds from *Ganoderma*, [22–25] including polysaccharides, triterpenes, sterols, and a few farnesyl phenolic substances [26–30].

A chemical investigation on the edible fruiting bodies of *G. sinense* led to the isolation of eight farnesyl phenolic compounds (1–8) (Figure 1a) (the chromatography graphs and spectra see Supplementary Figures 1-33), including six new ones, ganosinensols E–J (1–6), and two known ones, ganosinensols C–D (7–8) [31]. All of these farnesyl phenols possess a coumaroyl substitution in their structures, which are rather unusual in nature. They were predicted to have the high binding affinity to MTH1 with more negative mfScores among all the compounds from the in-house database. The enzyme inhibition experiments and cell-based assays confirmed their inhibitory effects on MTH1. The cytotoxicities of 1–8 in normal human cells were also evaluated. The siRNA knockdown experiments and the CETSA were carried out to verify the interaction between compounds and MTH1 protein in intact cells.

**Figure 1:**
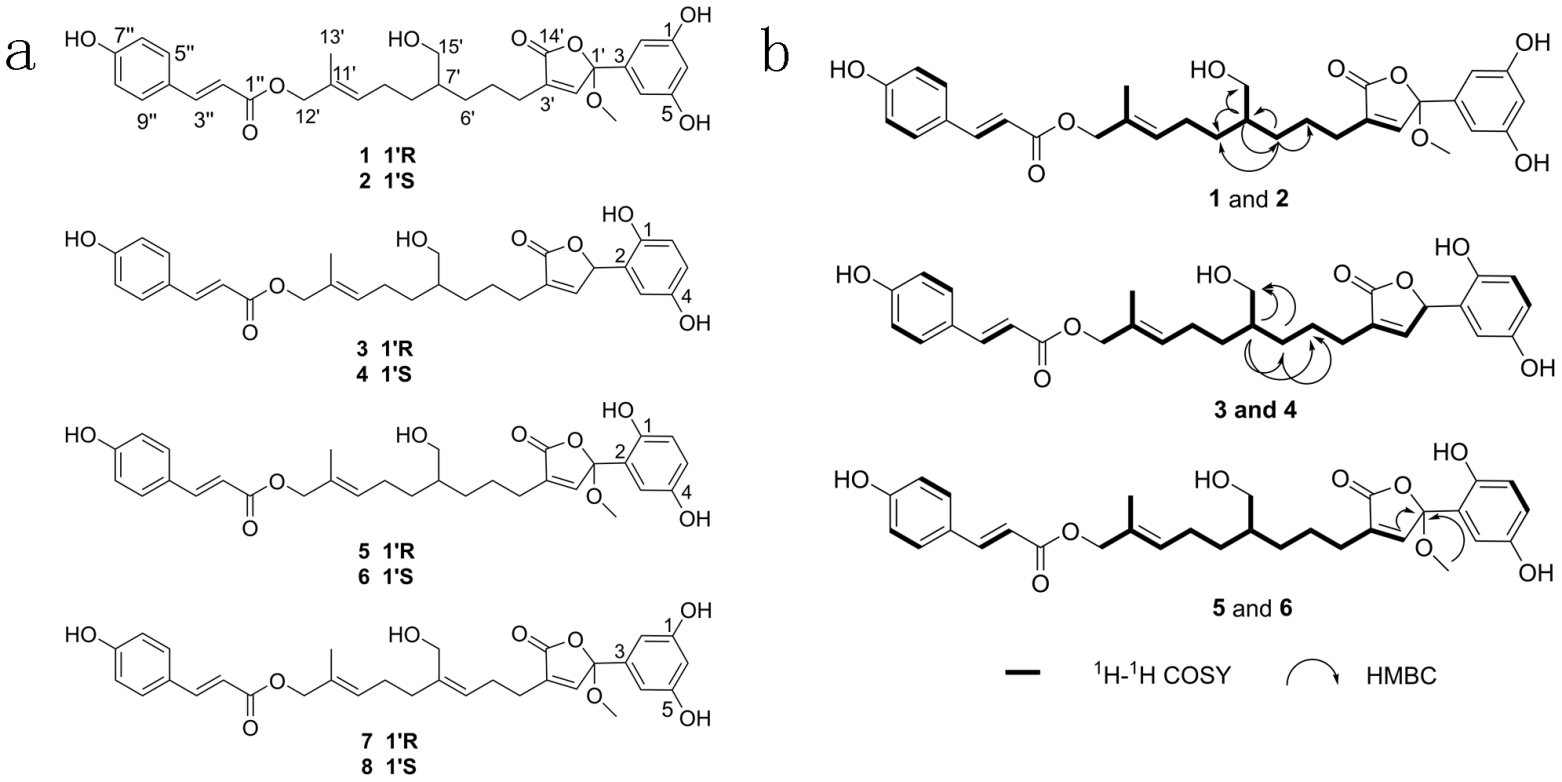
Chemical structures of the eight farnesyl phenolic compounds **(a)** Structures of compounds 1-8. **(b)** Key H^1^-H^1^ COSY and HMBC correlations of compounds 1-6.

## RESULTS

### Isolation and structure elucidation

Compounds 1 and 2 was assigned the molecular formula as C_31_H_36_O_9_ based on ^13^C NMR data and the [M + Na]^+^ at *m/z* 575.2260 (calcd 575.2257) ion in the positive HRESIMS. The ^1^H, ^13^C NMR (Table 1) and HSQC spectra suggested the presence of a 1,3,5-trisubstituted dihydroxybenzene moiety [*δ_H_* 6.94 (1H, br s), 6.67 (2H, br s)], a *p*-substituted hydroxybenzene structure [*δ_H_* 7.45 (2H, d, *J* = 8.4 Hz), 6.80 (2H, d, *J* = 8.4 Hz)], one methyl group, seven methylene groups, one oxygenated tertiary carbon, and two ester carbonyls. The ^1^H and ^13^C NMR data (Table 1) were consistent with those of 7 and 8, [31] except that the Δ^6',7'^ double bond (*δ_C_* 140.9/127.5 and *δ_H_* 5.24) was reduced (*δ_C_* 41.0/31.5 and *δ_H_* 1.47/1.31) in 1 and 2. C-5′–C-6′, C-7′–C-8′ and C-7′–C-15′ moieties were identified by the ^1^H-^1^H COSY spectrum. The HMBC correlations (Figure 1b) from H-6' (*δ_H_* 1.31) to C-5′/C-7′/C-8′, and H-7' (*δ_H_* 1.47) to C-6′/C-8′/C-15′, corroborated the hydration of Δ^6',7'^ double bond. The *E* configuration of Δ^10',11'^ double bond was assigned according to the NOESY correlation of H-10′ with H-12′.

**Table 1:** ^1^H and ^13^C NMR spectroscopic data for compounds 1-6*^a^*

Position	1 and 2*^b^*		3 and 4*^b^*		5 and 6*^c^*	
	*δ_C_*, type	*δ_H_* (*J* in Hz)	*δ_C_*, type	*δ_H_* (*J* in Hz)	*δ_C_*, type	*δ_H_* (*J* in Hz)
**1**	151.4, C		149.1, C		147.4, C	
**2**	118.5, CH	6.67, br s	123.6, C		122.0, C	
**3**	123.6, C		113.5, CH	6.47, d (2.8)	113.2, CH	6.87, d (2.8)
**4**	118.4, CH	6.67, br s	151.6, C		149.6, C	
**5**	149.4, C		117.4, CH	6.60, dd (8.6, 2.8)	117.0, CH	6.61, dd (8.4, 2.8)
**6**	114.8, CH	6.94, br s	117.4, CH	6.67, d (8.6)	117.1, CH	6.66, d (8.4)
**1'**	108.7, C		80.0, CH	6.23, br s	106.5, C	
**2'**	147.9, CH	7.40, br s	151.0, CH	7.35, br s	145.9, CH	7.38, d (1.0)
**3'**	136.7, C		133.7, C		134.9, C	
**4'**	26.3, CH_2_	2.27, t (6.8)	26.4, CH_2_	2.30, t (7.2)	24.8, CH_2_	2.20, t (7.6)
**5'**	25.9, CH_2_	1.59, m	26.1, CH_2_	1.61, m	24.2, CH_2_	1.49, m
**6'**	31.5, CH_2_	1.31, m	31.6, CH_2_	1.43, m	30.0, CH_2_	1.22, m
**7'**	41.0, CH	1.47, m	41.1, CH	1.50, m	39.4, CH	1.37, o
**8'**	31.7, CH_2_	1.40, m	31.7, CH_2_	1.34, m	30.3, CH_2_	1.36, o
**9'**	26.1, CH_2_	2.06, td (7.2, 7.2)	26.2, CH_2_	2.08, m	24.5, CH_2_	1.98, td (7.6, 7.2)
**10'**	130.8, CH	5.49, t (6.6)	130.8, CH	5.50, br s	129.1, CH	5.46, t (6.8)
**11'**	131.7, C		131.7, C		129.9, C	
**12'**	71.2, CH_2_	4.55, br s	71.2, CH_2_	4.55, br s	69.0, CH_2_	4.50, br s
**13'**	14.3, CH_3_	1.68, s	14.3, CH_3_	1.69, d (3.7)	13.7, CH_3_	1.61, s
**14'**	173.6, C		176.9, C		171.0, C	
**15'**	65.4, CH_2_	3.46, br d (3.7)	65.5, CH_2_	3.48, br d (4.8)	63.0, CH_2_	3.29, br d (4.4)
**1''**	169.3, C		169.3, C		166.4, C	
**2''**	115.3, CH	6.33, d (15.6)	115.4, CH	6.33, d (16.0)	114.0, CH	6.40, d (16.0)
**3''**	146.7, CH	7.60, d (15.6)	146.7, CH	7.60, d (16.0)	144.7, CH	7.55, d (16.0)
**4''**	127.3, C		127.3, C		124.9, C	
**5''9''**	131.3, CH_2_	7.45, d (8.4)	131.3, CH_2_	7.45, d (8.6)	130.3, CH_2_	7.54, d (8.4)
**6''8''**	117.0, CH_2_	6.80, d (8.4)	117.0, CH_2_	6.79, d (8.6)	115.8, CH_2_	6.78, d (8.4)
**7''**	161.4, C		161.4, C		160.0, C	
**OCH_3_**	52.3, CH_3_	3.27, s			51.0, CH_3_	3.16, s

*^a 1^*H NMR, 400 MHz; ^13^C NMR, 100 MHz. The assignments were based on HSQC and HMBC experiments. The ^1^H and ^13^C NMR data were identical for each pair of enantiomers.

*^b^* Measured in CD_3_OD.

*^c^* Measured in DMSO-*d*_6_.

**Figure 2:**
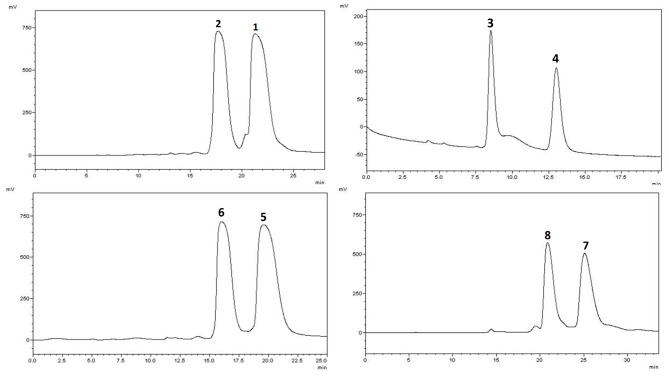
Chiral HPLC separation profiles of 1-8

The ECD and optical rotation data ([ α ]D20−5), as well as the completely identical NMR spectroscopic data indicated that the mix of 1 and 2 was an enantiomeric mixture. Subsequent chiral resolution on Chiralpak IE liquid chromatography afforded the enantiomers 1 and 2 in a ratio of approximately 1:1 (Figure 2). In the ECD spectrum, negative and positive Cotton effects at 210 and 230 nm suggested that the absolute configuration of C-1′ of 1 should be *R*, correspondingly, 2 was 1′*S*. On the basis of these findings, the structures of compounds 1 and 2 were defined as (5*R*)-3-{9-[(*E*)-coumaroyl]-4-hydroxymethyl-8-methyl-7-nonene}-5-(3,5-dihydroxyphenyl)-5-methoxy-2(5*H*)-furanone and (5*S*)-3-{9-[(*E*)-coumaroyl]-4-hydroxymethyl -8-methyl-7-nonene}-5-(3,5-dihydroxyphenyl)-5-methoxy-2(5*H*)-furanone, and they were named ganosinensol E and ganosinensol F, respectively.

Compounds 3 and 4 were determined to have a molecular formula of C_30_H_34_O_8_, as deduced from ^13^C NMR spectroscopic and HRESIMS data [M + Na]^+^ at *m/z* 545.2153 (calcd 545.2151), implying 14 indices of hydrogen deficiency, and 2 mass units higher than the known compounds, ganosinensols A and B [31]. The ^1^H and ^13^C NMR data (Table 1) indicated that the Δ^6',7'^ double bond (*δ_C_* 139.9/124.6 and *δ_H_* 5.18) in ganosinensols A and B was saturated (*δ_C_* 41.1/31.6 and *δ_H_* 1.50/1.43) in 3 and 4, which was further determined by the HMBC correlations (Figure 1b) from H-6' (*δ_H_* 1.43) to C-5′/C-7′/C-15′, and H-7' (*δ_H_* 1.50) to C-5′/C-6′/C-15′. The NOESY correlation of H-10′ with H-12′ suggested that the geometry of Δ^10',11'^ double bond was *trans*.

The small optical rotation value and similar Cotton effects in the ECD spectrum of the mix of 3 and 4 compared with those of 1 and 2 inferred that it was likely an enantiomeric mixture. The chiral HPLC purification afforded the enantiomers 3 and 4 in a ratio of approximately 1:1 (Figure 2). The ECD spectrum of 3 showed negative and positive Cotton effects at 208 and 250 nm, respectively, suggesting a 1′*R*-configuration. Conversely, its enantiomer 4 should be 1′*S*. Therefore, the structures of compounds 3 and4 were concluded as (5*R*)-3-{9-[(*E*)-coumaroyl]-4- hydroxymethyl-8-methyl-7-nonene}-5-(2,5-dihydroxyphenyl)-2(5*H*)-furanone and (5*S*)-3-{9-[(*E*)-coumaroyl]-4-hydroxymethyl-8-methyl-7-nonene}-5- (2,5-dihydroxyphenyl)-2(5*H*)-furanone, and named ganosinensol G and ganosinensol H, respectively.

The molecular formula of 5 and 6 were established as C_31_H_36_O_9_, on the basis of ^13^C NMR spectroscopic and HRESIMS data [M + Na]^+^ at *m/z* 575.2261 (calcd 575.2257). The difference between 5/6 and 3/4 was the missing of *δ_C_* 80.0 (C-1') and the appearance of *δ_C_* 106.5/51.0 in 5 and 6 (Table 1), speculating the linkage of a methoxyl to C-1′ (*δ_C_* 106.5). The HMBC correlations (Figure 1b) of 1′-OCH_3_ (*δ_H_* 3.16) with C-1′ (*δ_C_* 106.5), and H-2' (*δ_H_* 7.38) with C-1′ (*δ_C_* 106.5), confirmed the above supposition. The geometry of Δ^10',11'^ double bond was determined as *trans* based on the NOESY correlation of H-10′ with H-12′.

Although the mix of compounds 5 and 6 showed measurable optical rotations ([ α ]D20−10), its enantiomeric nature was demonstrated by chiral HPLC. The chiral HPLC purification afforded enantiomers 5 and 6 in a ratio of approximately 1:1 (Figure 2). The enantiomers also displayed typical antipodal ECD curves and opposite specific rotations. The absolute configurations of 5 and 6 were determined as 1′*R* and 1′*S*, respectively, using the same methods as described in 1 and 2. The combined data established the structures of 5 and 6 as (5*R*)-3-{9-[(*E*)-coumaroyl]-4-hydroxymethyl- 8-methyl-7-nonene}-5-(2,5-dihydroxyphenyl)-5-methoxy-2(5*H*)-furanone and (5*S*)-3- {[9-[(*E*)-coumaroyl]-4-hydroxymethyl-8-methyl-7-nonene}-5-(2,5-dihydroxyphenyl) -5-methoxy-2(5*H*)-furanone, which were named ganosinensol I and ganosinensol J, respectively.

### Structure based virtual ligand screening

**Figure 3:**
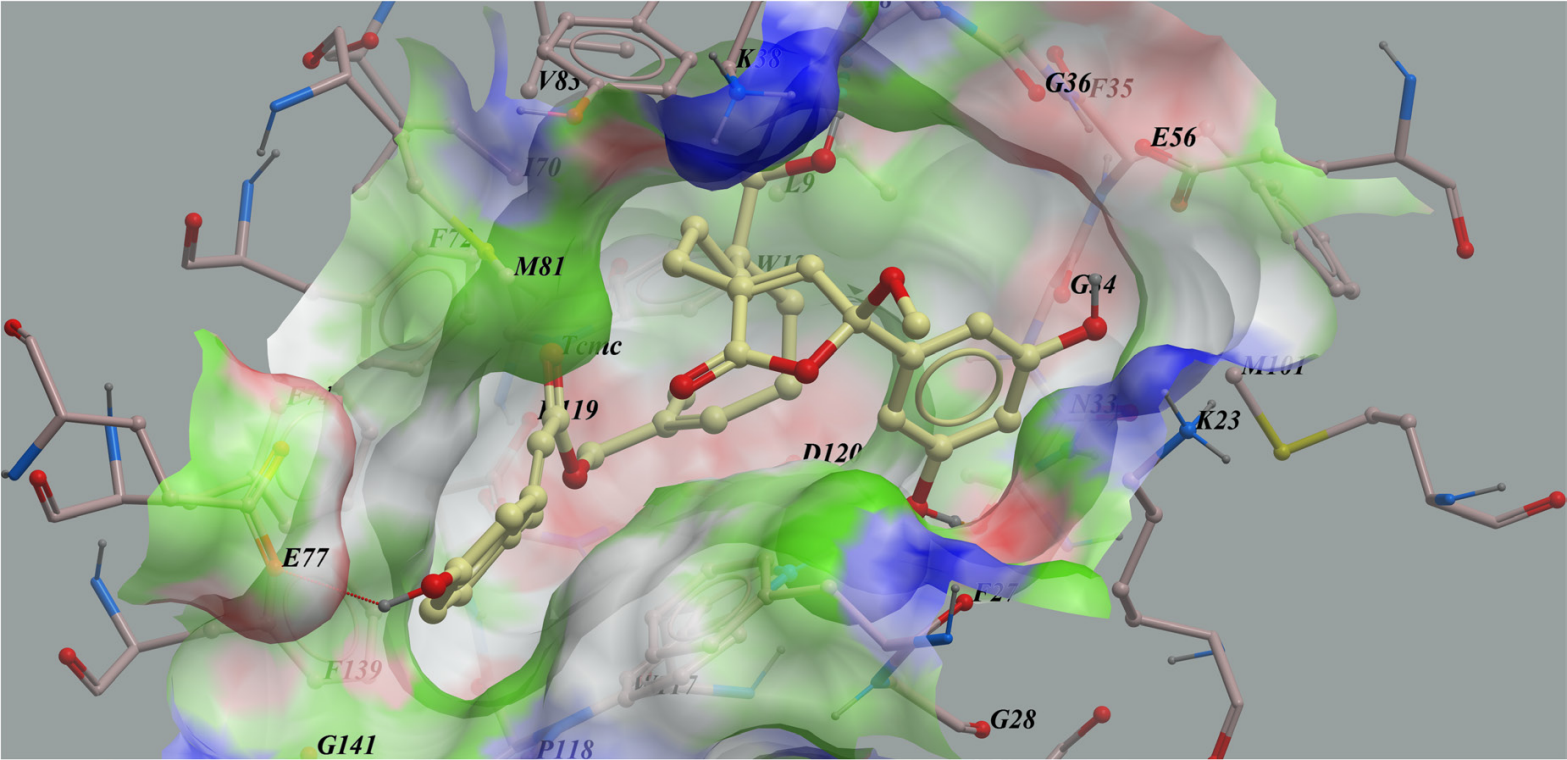
Low-energy binding conformations of compound 1 bound to MTH1 generated by virtual ligand docking Compound 1 depicted as the ball-and-stick model showing carbon (yellow), hydrogen (grey) and oxygen (red) atoms. Compounds were observed to occupy the active site of the enzyme and adopted a conformation similar to that of known inhibitors.

The eight isolated farnesyl phenolic compounds (1–8) were screened against the MTH1 model *in silico* based on its X-ray structure (PDB code: 4C9X) [8] during the preliminary evaluation. Compounds with lower calculated binding energies were considered to have higher binding affinities for the target. The results predicted that these farnesyl phenolic compounds exhibited the high binding affinity to MTH1 with negative mfScores of -163.4~-215.1 (Table 2). From the generated docking model, these kind of compounds were adopted a folded conformation, which occupied the active site of the enzyme, hydrogen bonds were predicted between 15′-hydroxyl group and carbonyl group of Thr8, 1-hydroxyl group and carbonyl group of Asn33, as well as between 7′′-hydroxyl group and carboxyl group of Glu77. Also, compound 1 formed key hydrophobic interactions between 1′-benzene ring and side chain of Lys23, and between furan ring and Tyr7. The π-π-π stacking interaction formed by 3′′-benzene ring with Phe27 and Phe74, further strengthens the binding (Figure 3).

**Table 2:** Binding affinity and inhibitory activities of 1-8 against MTH1 enzyme *in vitro*

No.	mfScores (kcal/mol)	MTH1	
		Kd (μM)	IC_50_ (μM)
1	-215.1	4.710±0.686	10.92±1.04
2	-182.9	2.380±0.663	10.95±1.03
3	-180.4	246.0±38.7	22.90±1.04
4	-187.5	4.630±0.519	22.89±1.07
5	-173.2	296.0±13.3	16.33±1.03
6	-163.4	113.0±25.4	8.62±1.03
7	-200.8	103.0±32.1	28.48±1.05
8	-202.3	210.0±42.7	26.60±1.03
(*S*)-crizotinib*^a^*	N.P.*^b^*	0.018±0.002	0.88±1.51

*^a^* (*S*)-crizotinib was used as positive control.

*^b^* N.P. represents mfScores of (*S*)-crizotinib was not performed.

### Specific binding with MTH1 *in vitro*

**Figure 4:**
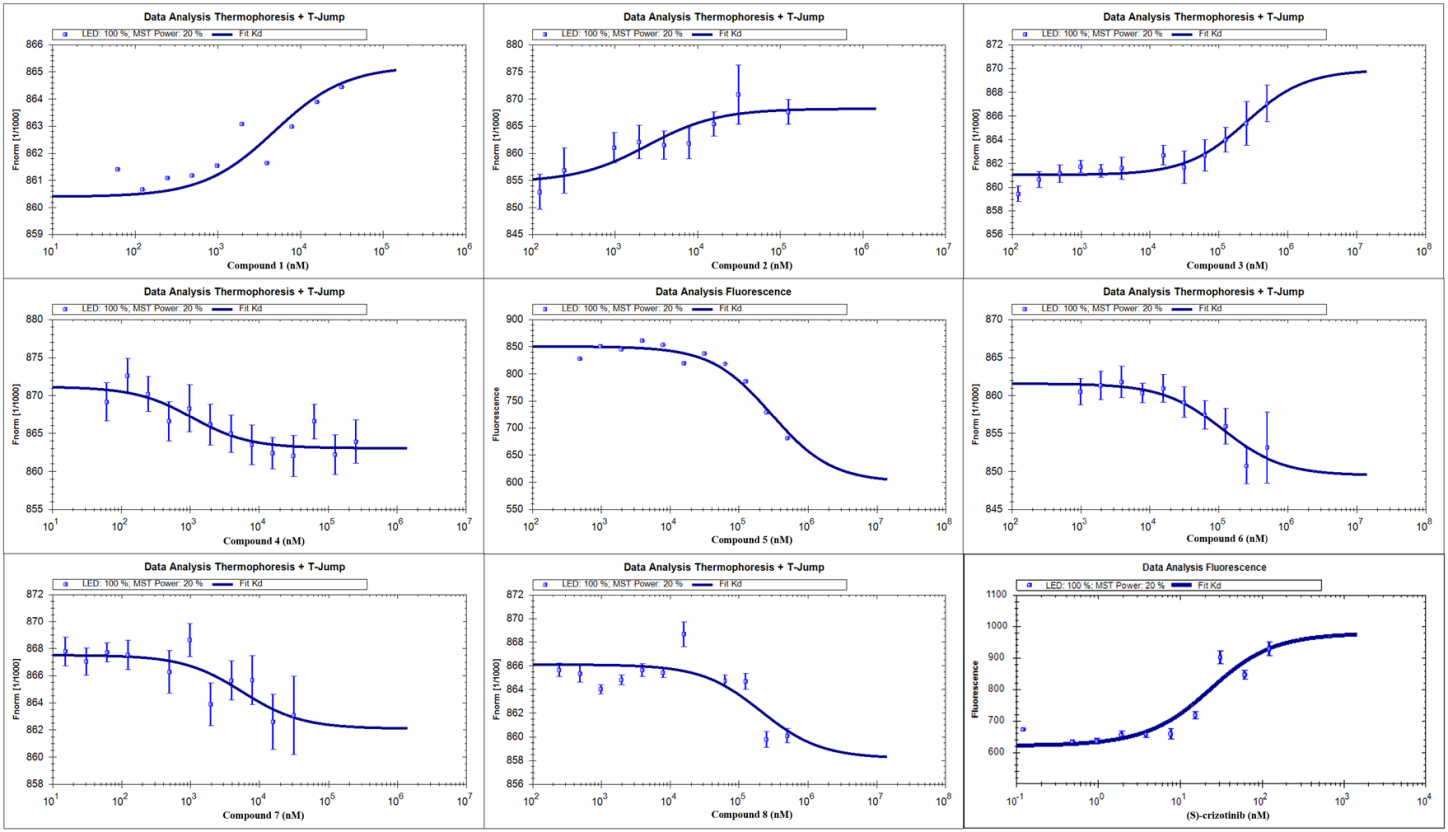
MST assay confirmed the specific binding of compounds 1-8 and (*S*)-crizotinib to MTH1 Measurement of affinity between compounds with MTH1 was carried out by MST in triple. The dissociation constant Kd values were automatically calculated by using the NT Analysis software (Nano Temper Technologies, München, Germany).

To validate the finding of the virtual ligand screening, we expressed the recombinant protein of human MTH1 [9]. Microscale thermophoresis method (MST) was employed to assay the binding affinity between the compounds and MTH1. This technology can quantify protein-protein interactions or protein-small molecule interactions with high sensitivity through detecting fluorescent changes of molecules during thermophoresis [32]. Among all the tested compounds, ganosinensols E (1) and F (2) exhibited the strongest binding with MTH1 (Table 2 and Figure 4). The equilibrium dissociation constants (Kd) of ganosinensols E (1) and F (2) were 4.71 and 2.38 μM, respectively. Further enzyme inhibition experiments showed that ganosinensols E (1) and F (2) could significantly inhibit the activity of MTH1 *in vitro* with IC_50_ of 10.92 and 10.95 μM, respectively (Table 2). Besides, compounds 3–8 also showed certain binding affinities to MTH1 and inhibitory activities (Table 2).

### Farnesyl phenolic compounds showed inhibitory effects on cancer cells and induced apoptosis

The growth inhibitory effects of the isolated farnesyl phenolic compounds (1–8) against carcinoma cell lines [human osteosarcoma cells (U2OS), human colorectal carcinoma cells (SW620), human ES-2 ovarian carcinoma cells (ES-2)] and normal tissue cell lines [human hepatic cells (Lo2), African green monkey kidney vero cells (Vero)] were further investigated using CCK8 assay method (Table 3). All of these compounds showed potential antitumor activity against the tested cell lines with IC_50_ values of 45.0~111.4 μM, slightly weaker than that of the positive control (*S*)-crizotinib. Noteworthily, the much higher IC_50_ values (most of them more than 300 μM) of these compounds on normal tissue cells indicated that they induced cell growth inhibition obviously in tumor cells and without significant cytotoxicity toward normal human cells, showing their better selectivity than (*S*)-crizotinib intracellularly.

**Table 3:** IC_50_ values*^a^* (μM) of compounds 1-8 against carcinoma and normal tissue cell lines

	Carcinoma cell lines			Normal tissue cell lines	
No.	U2OS	SW620	ES-2	Lo2	Vero
1	111.40±1.29	45.02±1.23	55.50±1.08	150.30±1.09	>300
2	54.24±1.10	103.80±1.07	76.78±1.09	>300	260.40±1.16
3	73.19±1.24	70.93±1.08	90.58±1.12	>300	>300
4	66.44±1.11	56.46±1.15	64.24±1.28	279.10±2.17	>300
5	64.40±1.08	61.80±±1.06	80.13±3.56	>300	>300
6	57.79±1.07	53.72±1.06	37.26±3.56	>300	>300
7	96.30±1.13	100.70±1.07	74.45±1.17	>300	>300
8	95.36±1.05	55.21±1.16	60.88±1.34	>300	>300
(*S*)-C*^b^*	32.28±1.28	17.50±1.11	9.09±1.06	26.01±1.20	16.69±1.15

*^a^* IC_50_ values of the isolated compounds were obtained from the mean OD values of the triplicate tests versus the drug concentration curves.

*^b^* (*S*)-C represents (*S*)-crizotinib, which was used as positive control.

**Figure 5:**
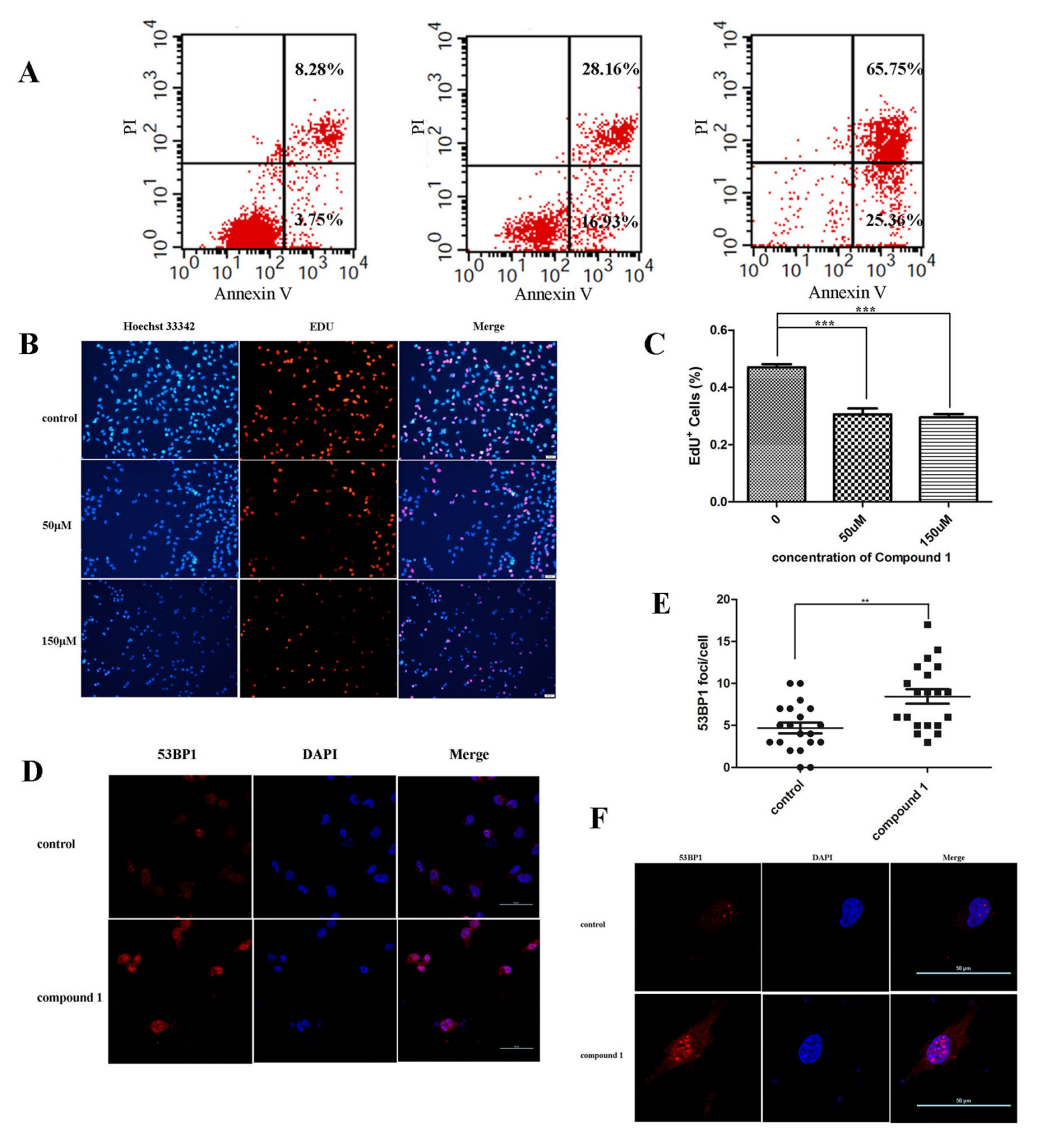
Compound 1 induced DNA damage and hindered the replication of DNA in cancer cells causing cell apoptosis **(A)** SW620 cells were treated with 0, 50 or 150 μM of compound 1 and then processed for FACS by using Annexin V/propidium iodide staining. **(B, C)** Immunofluorescence staining analysis of EDU^+^ and quantification of EDU^+^ in SW620 cells treated with 0, 50 or 150 μM of compound 1. Stained graphs were observed through a fluorescence microscope (× 200 magnification). Bar = 20 μm. Data are shown as mean ± s.d. (n = 3). **(D, E, F)** Immunofluorescence staining analysis of 53BP1 and quantification of 53BP1 foci formation in SW620 treated for 24 h with 50 μM of compound 1. It was observed through a confocal microscope (× 400 magnification). Bar = 50 μm Data are shown as mean ± s.d. (n = 3).

The apoptosis of cells induced by compounds was further investigated. SW620 cells were pretreated with series concentration of compound 1 for 24 hours. The number of apoptosis cells was quantified by flow cytometry. The ratio of apoptotic cells increased in compound 1 treated group in dose-dependent manner, with 45.09% and 91.11% SW620 cells apoptotic death at 50 μM and 150 μM of compound 1, respectively, compared with 12.03% for the control group (Figure 5A).

### Compound 1 induced DNA damage and hindered the replication of DNA in cancer cells

EdU cell proliferation assay and 53BP1 foci were performed here, demonstrating that farnesyl phenolic compounds could induce DNA damage and hinder the replication of DNA in cancer cells. EdU cell proliferation assays were carried out to detect the effects of compounds on DNA replication. EdU (5-Ethynyl-2- deoxyuridine), a thymidine analog, can penetrate into the replicating DNA molecule. Based on the specific response of EdU and fluorescent dyes, DNA replication activity could be detected. Results showed that the percentage of EdU^+^ cells significantly deceased when treated by compound 1 at theconcentration of 50 μM or 150 μM compared with the control (Figure 5B and 5C, P<0.001). The phenomenon proved precisely that compound 1 caused disruption of DNA replication (Figure 5B and 5C).

Meanwhile, MTH1 could prevent the mispairing of bases during replication and transversion mutations [5,6]. Presumably, its inhibitors could cause DNA damage. P53 binding protein 1 (p53-binding, protein 1, 53BP1) is an important regulatory factor in response to double strand breaks. The results showed that 53BP1 was significantly aggregated after treated with compound 1 compared to control, indicating the damage of DNA (Figure 5D-5F, P<0.01).

### Compound 1 interacted with MTH1 in cells

**Figure 6:**
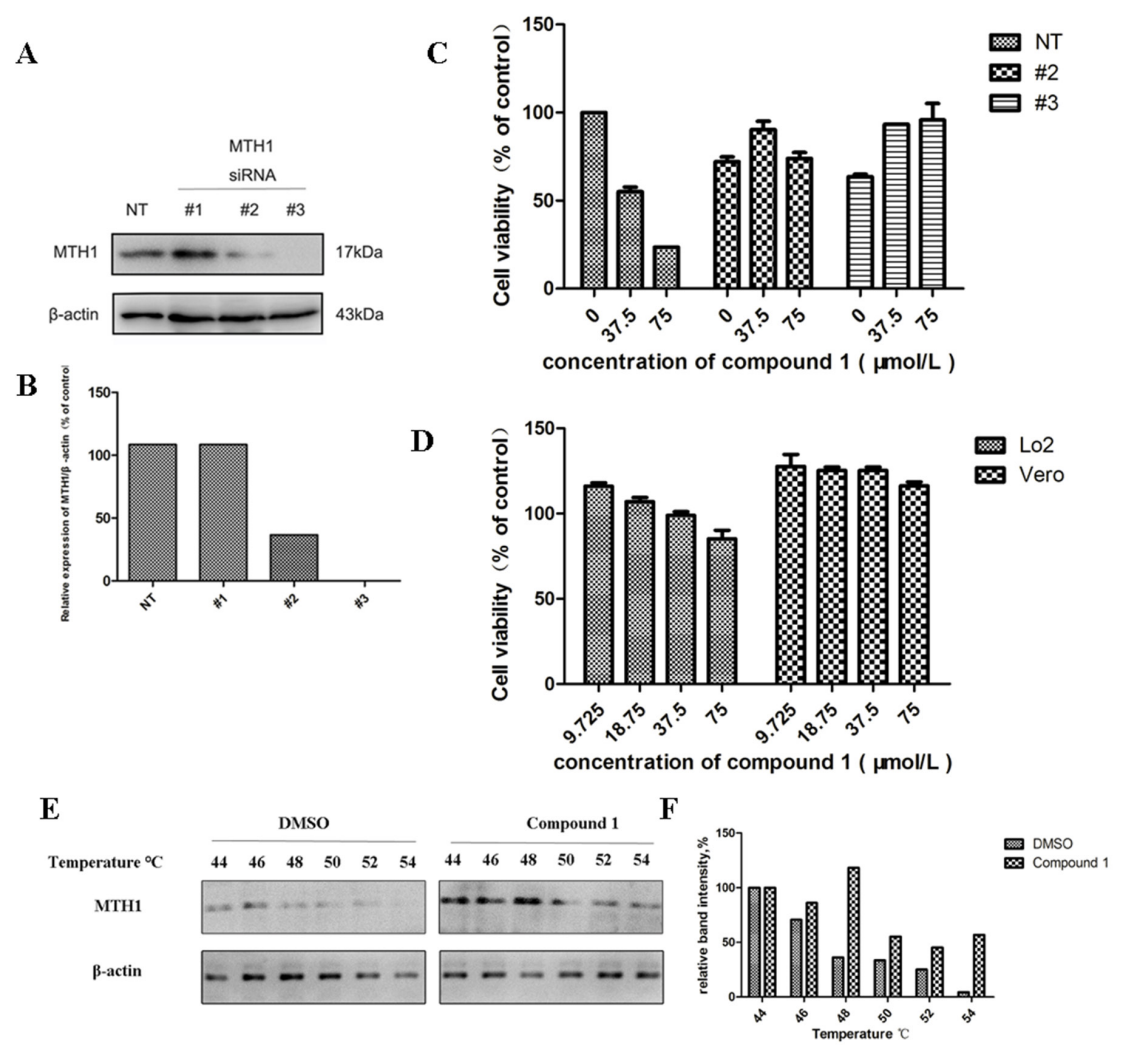
The specific binding of compound 1 to the MTH1 protein leads to cell growth inhibition **(A, B)** Western blot analysis and optical density analysis for the expression of MTH1 for the siRNA transfected cells. MTH1 siRNA #2 and #3 were both knocked down in SW620 cells compared with the non-target siRNA (NT). **(C)** The effects of compound 1 on the growth of MTH1 siRNA (#2 and #3) and non-targeting siRNA transfected cells were assessed by CCK8 assay. Results suggested that anticancer activities induced by compound 1 were dependent on the MTH1 in cells. **(D)** The effects of compounds 1 on normal tissue cell lines [human hepatic cells (Lo2), African green monkey kidney vero cells (Vero)]. **(E, F)** Cellular thermal shift assay (CETSA) confirmed the binding capacity of compound 1 with MTH1 at the cellular level. It is predictable that the specific binding of compound 1 to the MTH1 protein in SW620 cells.

In order to study whether the cytotoxicity of the farnesyl phenolic compounds was due to the inhibition of MTH1, the expression of endogenous MTH1 was knocked down in cancer cells. SW620 cell lines were transfected with MTH1 siRNA (#1, #2 and #3), [2] while the control was transfected with non-targeting siRNA (NT). The expression levels of MTH1 were measured by Western blot assay (Figure 6A and 6B), both siRNA #2 and #3 knocked down expressions of MTH1 in SW620 cells. The effects of compound 1 on the growth of MTH1 siRNA (#2 and #3) and non-targeting siRNA transfected cells were assessed by CCK8 assay. Results indicated that cells infected with MTH1 siRNA were resistant to the growth inhibition caused by compound 1 compared with non-targeting siRNA transfected cells (Figure 6C). Just as normal cells which do not need MTH1 (Figure 6D), survival cancer cells infected with MTH1 siRNA were insensitive to compound 1. These findings suggested that inhibitory activities induced by compound 1 were dependent on the MTH1 expression.

The cellular thermal shift assay (CETSA) was recently developed to further confirm the interaction between the target and the compound *in vitro* by Western blot assay. CETSA was applied based on the principles that the ligand binding gave rise to the thermal stabilization of target proteins. The thermal stability of MTH1 in SW620 cells was tested at the temperature range of 44-54°C after the pretreatment of compound 1 for 12 hours (Figure 6E and 6F). Correspondingly, the control cells were treated with DMSO. The results showed that MTH1 protein was still clearly detectable for the group pretreated with compound 1, but not for the DMSO treated group. Compared with the control group, the thermal stability of MTH1 protein in compound 1 pretreated cells was better under the same temperature condition. This suggested that the specific binding of compound 1 to the MTH1 protein in SW620 cells.

## DISCUSSION

In summary, this paper describes the isolation, chiral resolution and structure elucidation of four pairs of new farnesyl phenolic stereo isomers (1–8), from the fruiting bodies of *G. sinense*. The absolute configurations of C-1′ in the new enantiomers 1–6 were assigned by ECD spectra. The farnesyl phenols possessing a coumaroyl substitution have rarely been encountered in nature [31].

Structure-based virtual ligand screening was applied to discover small-molecule MTH1 inhibitors in early stage. As a result, the eight farnesyl phenols were found from a small in-house database of natural products through the above method. MST assays validated the results of virtual screening and quantitatively measured the affinity between candidates (compounds 1–8) and MTH1 protein. Subsequently, enzymatic and cell-based assays confirmed ganosinensols E (1) and F (2) as a potential inhibitor of MTH1.

The dysfunctional redox regulation led to the production of reactive oxygen species, causing damage to DNA and free dNTPs. The MTH1 protein that could clean up oxidized dNTP such as 8-oxo-dGTP or 2-OH-dATP, prevents incorporation of damaged bases during DNA replication to ensure the survival of cancer cells [4, 8]. Nevertheless, the dysfunctional redox regulation was not observed in normal cells. Thus, MTH1 protein played an important role to maintain tumor cell survival, while it was dispensable for the growth of normal cells. Structure-based virtual ligand screening and MST assays confirmed the specific binding between MTH1 protein and farnesyl phenolic compounds. In agreement with the prediction, the cytotoxicity assay results showed the different effects of farnesyl phenolic compounds on normal cells and cancer cells. These farnesyl phenolic compounds could inhibit the growth of various human cancer cells, while no obvious cytotoxicity was found on normal tissue cell lines, showing their features of selectivity and security as a potential candidate drug. And there was no notable difference on antitumor activity between each pair of stereo isomers, showing that the configuration of the chiral center is not vital for their activity. All these tend to imply that farnesyl phenolic compounds, especially for ganosinensols E (1) and F (2), are at least good lead compounds for designing novel MTH1 inhibitors. And it was also found that compound 1 induced DNA damage and hindered the replication of DNA in SW620 cells. Furthermore, siRNA knock down experiments and the CETSA were carried out to confirm the interaction between compound 1 and MTH1 in SW620 cells. Results implied that the farnesyl phenolic compounds specifically targeted MTH1 proteins in cells. Moreover, especially for siRNA knock down experiments, the toxic effect of the compound 1 was reduced after knockdown of MTH1 in cells. It did prove that the interaction between compound 1 and MTH1 was critical for its cytotoxicity. The project also suggested that the virtual ligand screening method is a useful tool to discover active lead compounds from natural products.

In this study, we chose (*S*)-crizotinib as the positive control for following reasons. (*S*)-crizotinib, was an effective inhibitor of MTH1 according to the relevant research [4, 8]. (*S*)-crizotinib was proved to be a MTH1 inhibitor by more technical means (MTH1 catalytic activity inhibition assays, ITC assays, etc) compared to other inhibitors, such as TH287 and TH588 with almost the similar binding affinity to MTH1 as (*S*)-crizotinib. In addition, (*S*)-crizotinib was the ligand of the co-crystal structure of MTH1 (PDB code: 4C9X) which was chosen as the template to generate our model to perform virtual ligand screening. This allowed the consistence for our research. Therefore, (*S*)-crizotinib is more reliable and suitable as the positive control compared to other chemical inhibitors.

While some recent articles reported that MTH1 may not be an independent anti-cancer target, [33–35] there is no doubt that MTH1 is a target of cellular antioxidative defenses [1, 36–38]. For both normal and cancerous cells, it is not uncommon that different cells have different sensitivities to MTH1 inhibitor. There were many factors, which influence effects of MTH1 inhibitors on the growth of cancer cells. For cancer cells with strong anti-oxidative defense, MTH1 inhibitor has no cytotoxicities. In contrast, MTH1 inhibitors have strong killing potency for cancer cells that have weak resistance to oxidative stress. It was reported that the disruption of MTH1 increased telomere dysfunctions and caused cancer cell deaths [1]. Thus cancer cells with shorten telomere would be more sensitive to MTH1 inhibition and the oxidative stress [1]. The inhibition or disruption of MTH1 is sure to reduce the cell's ability to cope with oxidative stress, especially for cancer cells [1–4, 36]. MTH1, which protects cancer cells from the oxidative-stress-induced damage of dNTP pools, is a promising target for designing effective anti-cancer drugs with low toxicities [1–4, 36–38].

As far as we know, farnesyl phenolic compounds are the first kind of natural products reported to exhibit MTH1 inhibitory activities. Therefore, this kind of compounds and their derivatives can be considered as lead compounds targeted MTH1 for drug development. Our work provides a reliable method to study the action mechanism of traditional Chinese medicine, and contributes to enrich structure scaffolds of MTH1 inhibitors. In our future study, the structural modifications and more detailed anti-tumor action mechanisms of farnesyl phenolic compounds will be further explored.

## MATERIALS AND METHODS

### General experimental procedures

Optical rotations were measured with a PerkinElmer 241 polarimeter (Perkin-Elmer, Waltham, MA, USA). UV spectra were recorded on a Shimadzu UV 2201 UV-VIS recording spectrophotometer (Shimadzu Corporation, Kyoto, Japan). ECD spectra were determined on a Bio-Logic Science MOS-450 spectrometer (Bio-Logic, Claix, France). IR (4000−400 cm^−1^) spectra (KBr disks) were recorded on a Bruker IFS 55 spectrometer (ßBruker Optics, Ettlingen, Germany). NMR experiments were performed on Bruker ARX-400 or AV-600 spectrometers (Bruker Biospin, Fallanden, Switzerland). Chemical shifts are stated relative to TMS and expressed in *δ* values (ppm), with coupling constants reported in Hz. HRESIMS were obtained on an Agilent 6210 TOF mass spectrometer (Palo Alto, USA). Silica gel GF254 prepared for TLC and silica gel (200-300 mesh) for column chromatography (CC) were obtained from Qingdao Marine Chemical Factory (Qingdao, China). Sephadex LH-20 was a product of Pharmacia (Amersharm, Sweden). Octadecyl silica gel was purchased from Merck Chemical Company Ltd (Darmstadt, Germany). RP-HPLC separations were conducted using a LC-6AD liquid chromatograph with a YMC Pack ODS-A column (250 × 20 mm, 5μm, 120 Å) and SPD-10AVP UV/vis detector (Shimadzu, Kyoto, Japan). Chiral-HPLC separations were conducted using a LC-6AD liquid chromatograph with a Daicel Chiralpak IE column (250 × 4.6 mm, 5μm; Daicel, Japan) and SPD-10AVP UV/vis detector. All reagents were HPLC or analytical grade and were purchased from Tianjin Damao Chemical Company (Tianjin, China). Spots were detected on TLC plates under UV light or by heating after spraying with anisaldehyde-H_2_SO_4_ reagent.

### Plant material

The fruiting bodies of *Ganoderma sinense* Zhao. Xu et Zhang were purchased from the Xunwu Prefecture of Jiangxi Province, People's Republic of China, and authenticated by Prof. Weining Wang at the Liaoning Institute for food and drug control. A voucher specimen (LZ-02-010) has been deposited in the same institution.

### Extraction and isolation

The fruiting bodies of *Ganoderma sinense* Zhao. Xu et Zhang (9.5 kg) were cut into approximately 2 cm pieces and extracted with 95% EtOH (100 L × 2 h × 2, 40-50°C). The resulting extract (233.6 g) was concentrated *in vacuo*, suspended in H_2_O (5 L), and partitioned successively with cyclohexane, EtOAc, and *n*-BuOH (5 L × 3). After evaporation of EtOAc *in vacuo*, the residue (81.4 g) was subjected to silica gel CC (10 × 80 cm; 200-300 mesh, 500 g) eluted with CH_2_Cl_2_/MeOH (100:1, 50:1, 20:1, 10:1, 5:1, 2:1, 1:1, and 0:1 v/v) to obtain eleven fractions (E1−E11), which were combined according to TLC analysis. Fr. E5 (26.3 g) was chromatographed on a silica gel CC (6 × 80 cm; 200-300 mesh, 300 g) and eluted with a gradient of increasing acetone (0–100%) in CH_2_Cl_2_, to produce five fractions (E51−E55). Separation of E53 (6.8 g) on a reversed-phase C18 silica gel CC (2.5 × 30 cm; 200-300 mesh, 100 g) eluted with MeOH/H_2_O (10:90, 30:70, 50:50, 70:30, and 100:0 v/v) yielded fractions E531 to E535. Fraction E532 (500.2 mg) was separated further by Sephadex LH-20 CC (2 × 80 cm) by elution with MeOH to give four subfractions (E5321-E5324). Fr. E533 (1.1 g) was subjected to Sephadex LH-20 CC (2 × 80 cm) by elution with MeOH to yield four subfractions (E5331-E5334). E5334 (307.9 mg) was purified by preparative HPLC (65% MeOH/H_2_O, flow rate 6.0 mL/min) to afford 7/8 (36.1 mg, *t*_R_ 70 min) and 1/2 (110.5 mg, *t*_R_ 80 min). Fr. E534 (754.5 mg) was further chromatographed via Sephadex LH-20 CC (2 × 80 cm) using MeOH to give five subfractions (E5341-E5345). Subfraction E5344 (334.9 mg) yielded 3/4 (34.4 mg, *t*_R_ 55 min) and 5/6 (20.4 mg, *t*_R_ 60 min) upon separation on a preparative HPLC (45% MeCN/H_2_O, flow rate 6.0 mL/min).

Compounds 1/2, 3/4, 5/6 and 7/8 are enantiomeric mixtures, indicating by their optical rotations, ECD spectra and identical NMR data, which were subjected to chiral HPLC (Daicel Chiralpak IE, flow rate: 0.8 mL/min) to yield (*S*)-2 (5.8 mg, *t*_R_ = 18.0 min) and (*R*)-1 (5.7 mg, *t*_R_ = 22.0 min) (*n*-hexane/isopropanol, 75:25), (*R*)-3 (5.9 mg, *t*_R_ = 8.5 min) and (*S*)-4 (5.5 mg, *t*_R_ = 13.0 min) (*n*-hexane/isopropanol, 65:35), (*S*)-6 (4.1 mg, *t*_R_ = 16.5 min) and (*R*)-5 (4.4 mg, *t*_R_ = 20.0 min) (*n*-hexane/isopropanol, 75:25), (*S*)-8 (4.9 mg, *t*_R_ = 20.5 min) and (*R*)-7 (5.0 mg, *t*_R_ = 25.0 min) (*n*-hexane/isopropanol, 75:25).

### Spectroscopic data of the isolated compounds

#### Ganosinensols E (1) and F (2)

pale yellow oil (MeOH); {[ α ]D2014 (*c* 0.1, MeOH); CD (MeOH) nm (Δ*ε*) 210 (-1.51), 230 (+8.92); (*R*)-1}; ([ α ]D20−47 (*c* 0.1, MeOH); CD (MeOH) nm (Δ*ε*) 214 (+2.41), 232 (-7.46); (*S*)-2}; UV (MeOH) *λ*_max_ (log *ε*) 312 (4.46) nm; IR (KBr) *ν*_max_ 3383, 2938, 1755, 1682, 1605, 1455, 1170, 1025, 833 cm^−1^; ^1^H NMR (400 MHz, CD_3_OD) and ^13^C NMR (100 MHz, CD_3_OD) data, see Table 2 ; HRESIMS (positive) *m/z* 575.2260 [M + Na]^+^ (calcd for C_31_H_36_O_9_Na, 575.2257).

#### Ganosinensols G (3) and H (4)

pale yellow oil (MeOH); ([ α ]D20−68 (*c* 0.1, MeOH); CD (MeOH) nm (Δ*ε*) 208 (-29.59), 250 (-2.50); (*R*)-3};([ α ]D20+32 (*c* 0.1, MeOH); CD (MeOH) nm (Δ*ε*) 207 (+34.37), 249 (-0.92); (*S*)-4}; UV (MeOH) *λ*_max_ (log *ε*) 310 (4.55) nm; IR (KBr) *ν*_max_ 3363, 2940, 1740, 1691, 1605, 1453, 1170, 1023, 833 cm^−1^; ^1^H NMR (400 MHz, CD_3_OD) and ^13^C NMR (100 MHz, CD_3_OD) data, see Table 1 ; HRESIMS (positive) *m/z* 545.2153 [M + Na]^+^ (calcd for C_30_H_34_O_8_Na, 545.2151).

#### Ganosinensols I (5) and J (6)

pale yellow oil (MeOH); {[*α*]^20^_D_38 (*c* 0.1, MeOH); CD (MeOH) nm (Δ*ε*) 210 (-1.63), 231 (+10.12); (*R*)-5}; {[ α ]D20−18 (*c* 0.1, MeOH); CD (MeOH) nm (Δ*ε*) 211 (+1.36), 231 (-7.30); (*S*)-6}; UV (MeOH) *λ*_max_ (log *ε*) 313 (4.37) nm; IR (KBr) *ν*_max_ 3427, 2935, 1747, 1681, 1605, 1453, 1168, 1025, 833 cm^−1^; ^1^H NMR (400 MHz, DMSO-*d*_6_) and ^13^C NMR (100 MHz, DMSO-*d*_6_) data, see Table 2 ; HRESIMS (positive) *m/z* 575.2261 [M + Na]^+^ (calcd for C_31_H_36_O_9_Na, 575.2257).

### Structure based virtual ligand screening

ICM 3.8.2 modeling software on an Intel i7 4960 processor (MolSoft LLC, San Diego, CA) was used to identify possible drug candidates for MTH1 enzyme. For the structure-based virtual screening, ligands were continuously resiliently made docking with MTH1 which was represented in potential energy maps. Compounds were scored according to the internal coordinate mechanics (Internal Coordinate Mechanics, ICM). Conformational sampling was based on the Monte Carlo procedure [39] and finally the lowest-energy and the most favorable orientation of the ligand were selected.

### Protein expression and purification of MTH1

The gene of the human mutT homologue MTH1 was ligated into PET 28a vector (Novagen). After the recombinant plasmid was verified by sequencing, it was transformed into *E. coli* strain BL21 Star (Invitrogen) at 293K, which were grown in LB medium at 37°C to an OD600 (0.8–1.0) and induced by 0.4 mM isopropyl-D-thiogalactopyranoside (IPTG) at 18°C for 16 hours. Bacterial cells were lysed by ultrasonification on ice in buffer containing 100 mM Tris-HCl pH 8.8, 200 mM NaCl, 10% glycerol, 1% TritonX100, 5 mM β-mercaptoethanol. Soluble N-terminally hexa-histidine tagged MTH1 was bound to Ni-agrose affinity resin (Qiagen), washed with a buffer containing 20 mM Tris-HCl pH 8.8, 200 mM NaCl and 10 mM imidazole and eluted with a buffer containing 20 mM Tris-HCl pH 8.8, 250 mM NaCl, and 150 mM imidazole. The eluted protein was concentrated and diluted with a buffer containing 20 mM Tris-HCl pH 8.8, 250 mM NaCl and digested with thrombin for 12–15 h at 277 K. Cut MTH1 was purified by Ni-agrose affinity resin (Qiagen). The protein was further purified with anion exchange chromatography (GE Health), using a linear gradient of 10 mM to 1 M NaCl concentration and size exclusion chromatography (GE Health) at 20 mM Tris-HCl pH 8.8 and 200 mM NaCl [40].

### Microscale thermophoresis (MST) assay for *in vitro* binding affinity

Recombinant human MTH1 was labeled with the Monolith NT™ Protein Labeling Kit RED (Cat#L001) according to the supplied labeling protocol [32]. Labelled MTH1 was kept constant at 100 nM, while all samples tested were diluted in a 20 mM HEPES (pH 7.5) and 0.05 (v/v) % Tween-20. Compounds were diluted in 12 dilution steps covering the range from 500 μM to 200 nM. After 10 min incubation at room temperature about 37°C, samples were loaded into Monolith™ standard-treated capillaries and the thermophoresis was measured at 25°C after 30 min incubation on a Monolith NT.115 instrument (NanoTemper Technologies, München, Germany). Laser power was set to 20% using 30 seconds on-time. The LED power was set to 100%. The dissociation constant Kd values were fitted by using the NTAnalysis software (NanoTemper Technologies, München, Germany). The Kd value of the binding was obtained in two ways. The first way was calculated by thermophoresis curve when there was no fluorescent change during the initial fluorescence scanning. The second way was calculated from the initial fluorescence scanning curve when compounds bound in the sites which directly interfered the fluorescence. The appropriate way was employed to obtain Kd values of eight compounds and (*S*)–crizotinib.

### Enzyme inhibition assay

To identify the efficacy of the eight compounds, an enzyme inhibition assay was carried out. A series of compounds in DMSO were incubated with purified MTH1 protein (1.53 mg protein/mL, 6 μL) in 42 μL buffer containing 5 mM MgCl_2_ and 100 mM Tris-HCl (pH 8.5) at a temperature of 25°C. 30 min later, 8-oxo-dGTP (final conc. 25 μM, 6 μL) was added to the mixture, and reaction was processing at a temperature of 37°C and 200 rpm. Then the reaction was stop by the addition of 20 μL ice-cold Na_2_EDTA (50 mM). The reaction mixture was centrifugated at 15000 rpm at 4°C for 10 min. 20 μL of supernatant was injected on an HPLC column (Ultimate AQ-C18 RP-18e, 250 mm × 4.6 mm × 5 μm), and eluted isolate with 100 mM Na_2_HPO_4_-NaOH (pH 5.5)/MeOH 95:5 with flow rate 1 mL/min at column room of 30°C. For detection of the formed 8-oxo-dGMP, the peak integrated with wavelength of 293 nM [9]. The inhibition rates were calculated using the concentration ratios of 8-oxo-dGMP in tubes with or without various concentration compounds. Measurements were done in triplet, and IC_50_ values were calculated to compare the efficiency of various compounds.

### Cytotoxicity assay

Carcinoma cell lines [human osteosarcoma cells (U2OS), human colorectal carcinoma cells (SW620), human ES-2 ovarian carcinoma cells (ES-2)] and normal tissue cell lines [human hepatic cells (Lo2), African green monkey kidney Vero cells (Vero)] The U2OS, SW620, ES-2, Lo2 and Vero were obtained from ATCC (Manassas, VA, USA). The SW620 and Vero cells were cultured in Roswell Park Memorial Institute medium (RPMI), with 10% fetal bovine serum (Hyclone) and 1% penicillin-streptomycin (Sigma). Lo2, U2OS and ES-2 cells were grown in Dulbecco's modified eagle medium (DMEM), with 10% fetal bovine serum (Hyclone) and 1% penicillin-streptomycin (Sigma). All of these cells were cultured at 37°C in a humidified atmosphere with 5% CO_2_. For CCK8 assay cytotoxicity evaluation of compounds, all cells were separately plated at a density of 1 × 10^4^ cells/well in a 96 well plate. Cells were cultivated at 37°C in 5% CO_2_ atmosphere for 24 h, then medium in the wells was replaced with fresh medium containing compounds which were diluted in 8 dilution steps covering the range from 200 μM to 1.5625 μM. And DMSO was used as negative control. After 48 h incubation, 10 μL of CCK8 solution was added to each well. After 30 min, the optical density (OD) of each well was measured at wavelength of 450 nm on spectrophotometer. Data were corrected for background (no-cell control) and expressed as a percentage of the value for untreated cells. The IC_50_ values of the isolated compounds were derived from the mean OD values of the triplicate tests versus the drug concentration curves [41].

### Apoptosis analysis

Apoptotic cells were staining with Annexin-V-FITC and Propidium Iodide (PI) (Annexin V-FITC/PI double staining cell apoptosis detection kit) following the manufacturer's protocol. Fluorescence was recorded by flow cytometry using the FACSort (BD Bio sciences) [42].

### EdU cell proliferation assay

SW620 cells were pretreated with 0, 50, 150 μM of compound 1, respectively. Dissociated cells were handled with EdU cell proliferation kit (RiboBio) according to the manuscript by according to the manufacturer's instructions. Then the morphology was observed by fluorescence microscopy (Zeiss, OBSERVER D1/AX10 cam HRC) [43].

### Immunofluorescence

Cells which adhered to glass coverslips were treated with DMSO or compounds (50 μM) for 24 h. Then cells were washed with PBS and then fixed with 3% paraformaldehyde in PBS for 20 min at -20°C. Fixed cells were rinsed with PBS and incubated with penetrating agent (0.5% Triton-X-100) in PBS for 5 min. Next, cells were stained with an anti-53BP1 monoclonal antibody (abcam, diluted 1:250). Then the coverslips were rinsed with PBS at least 3 times andincubated with an Alexa Fluor 568 goat anti-rabbit IgG secondary antibody. After PBS rinsing, coverslips were stained with DAPI (sigma). Then the morphology was observed by confocal microscopy (Zeiss, OBSERVER D1/AX10 cam HRC) [8].

### Transfections and IC_50_ value determination

SW620 cells were seeded with 5 × 10^5^ cells/well in 6 well plates and were transfected at 60-70% confluence the next day using 200 nM MTH1 siRNA or NT siRNA (GenePharma) complexed with 2 μg/ml Lipofectamine™ 2000 (Invitrogen, Cat. No. 11668-030) following the protocol of manufacture. Cells were cultured in opti-MEM media (Gibco) during transfection. The three siRNA sequences were showed as below.

MTH1 siRNA #1 GACGACAG CUACUGGUUUC;

MTH1 siRNA #2 GAAAUUCCACGGGUACUUC;

MTH1 siRNA #3 CGACGACAGCUACUGGUUU.

After transfection for 6 hours, opti-MEM media were replaced with DMEM media. And 48 hours later, cells were washed with PBS and suspended with PBS containing 1mM PMSF, and followed by Western blot assay. The IC_50_ of compound 1 was retested using siRNA transfected cells [2, 44].

### Cellular thermal shift assay (CETSA)

CETSA was developed as a method to directly detect the binding capacity of compounds with targets at the cellular level, based on the principle of ligand-induced target protein stabilization. Briefly, SW620 cells cultured with 90% confluent in 100 × 20 mm tissue culture dishes were treated with media containing DMSO or compound 1 (30 μM) for 12 hours. After treatment, the cells were isolated with trypsin, collected by centrifugation, and then resuspended in PBS. The cell suspension was divided equally into 4 PCR tubes and heated to 44, 46, 48, 50, 52, and 54°C for 3 minutes, respectively. Subsequently, cells were analysed by Western blot assay [45–46].

### Western blot assay

The harvested cells were lysed with liquid nitrogen and the freeze-thaw cycles were repeated twice. The soluble proteins were separated from the cell pellet by centrifugation at 14,000 g for 20 min. Proteins was quantified by Bradford reagent (Bio-Rad, USA). The same amount of proteins (20-40 μg) were loaded onto 15% SDS-PAGE gels and transferred to polyvinylidene difluoride membranes (PVDF) (Millipore, USA) and analyzed using the MTH1-antibody (Proteintech) at a concentration of 1: 500. The level of protein expression in Western blot was quantified by optical density analysis using Image J software program (NIH) [42].

## SUPPLEMENTARY MATERIALS FIGURES AND TABLES



## Abbreviations

MTH1: human mutT homologue
CETSA: cellular thermal shift assay
ROS: reactive oxygen species
OD: the optical density
IPTG: isopropylβ-D-1-thiogalactopyranoside
MST: microscale thermophoresis
PMSF: phenylmethanesulfonyluoride
PVDF: Polyvinylidene difluoride.
