# Supplementary material for: Farnesyl phenolic enantiomers as natural MTH1 inhibitors from Ganoderma sinense
Source: Oncotarget. 2017 Sep 30;8(56):95865–79. doi: 10.18632/oncotarget.21430 (PMC5707067; doi:10.18632/oncotarget.21430)
Supplement: Supplementary file 1 [file oncotarget-08-95865-s001.pdf]

Farnesyl phenolic enantiomers as natural MTH1 inhibitors from *Ganoderma sinense*

SUPPLEMENTARY MATERIALS

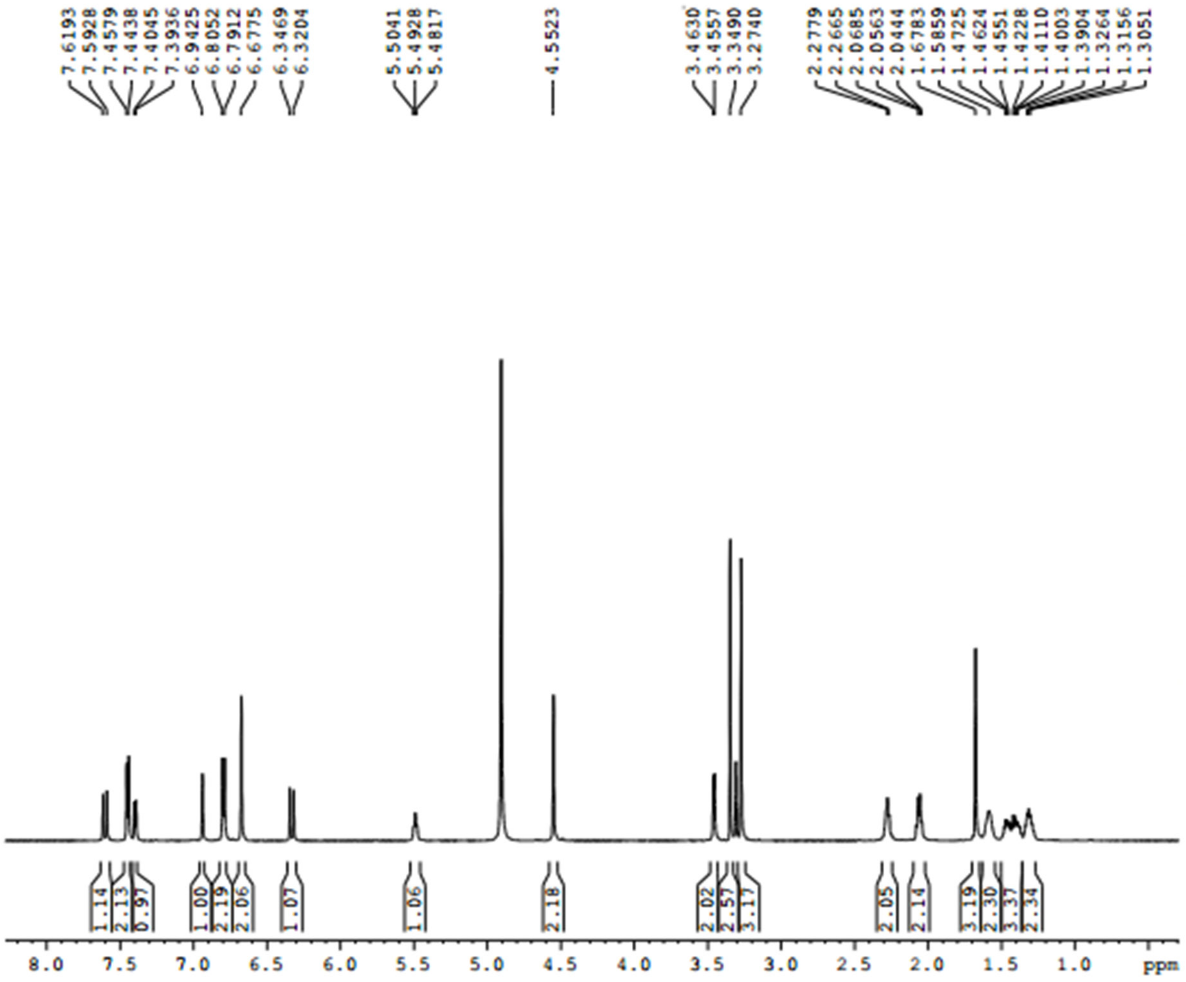

Supplementary Figure 1: ^{1}H NMR (400 MHz) spectrum of 1 and 2 in CD_3OD.

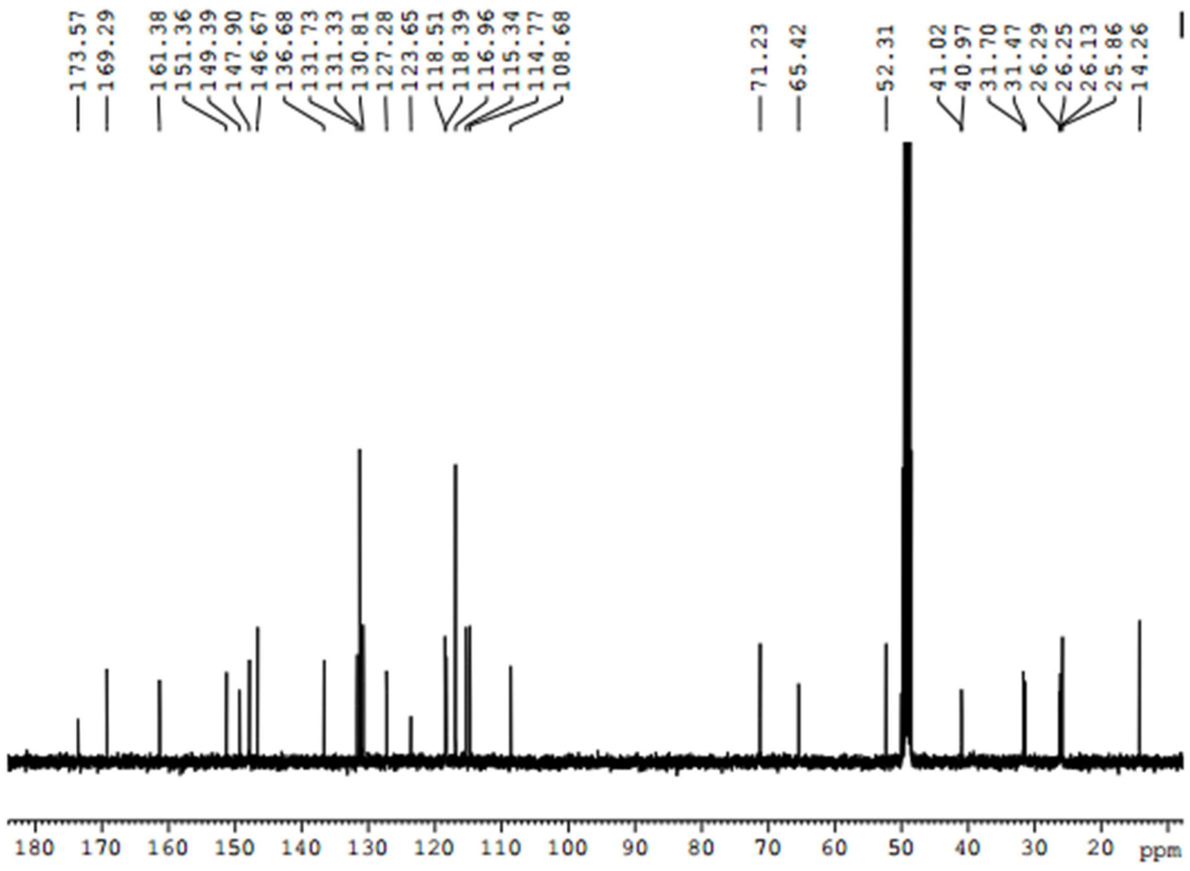

Supplementary Figure 2: **^{13}C NMR (600 MHz) spectrum of 1 and 2 in CD_3OD.**

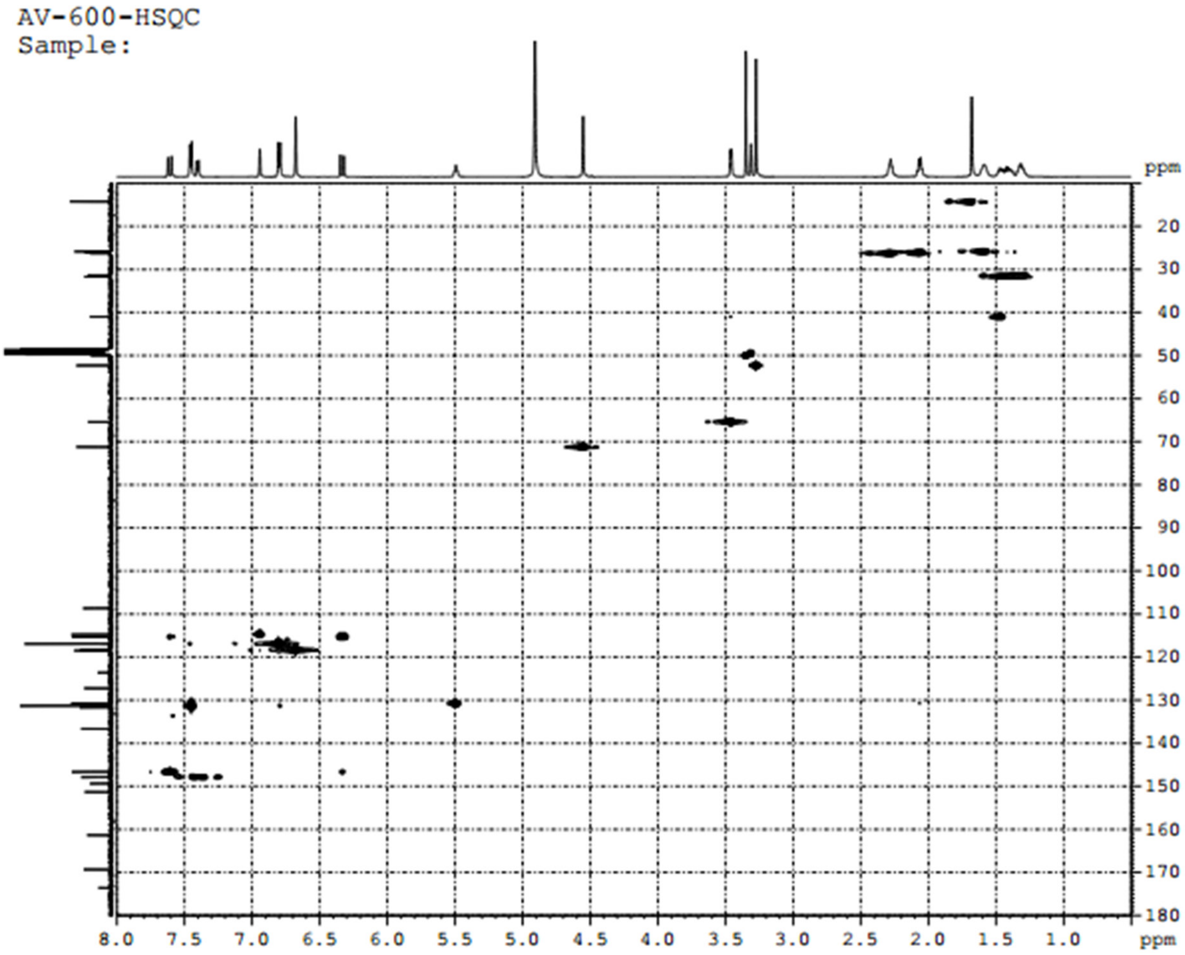

Supplementary Figure 3: HSQC spectrum of 1 and 2 in CD_3OD.

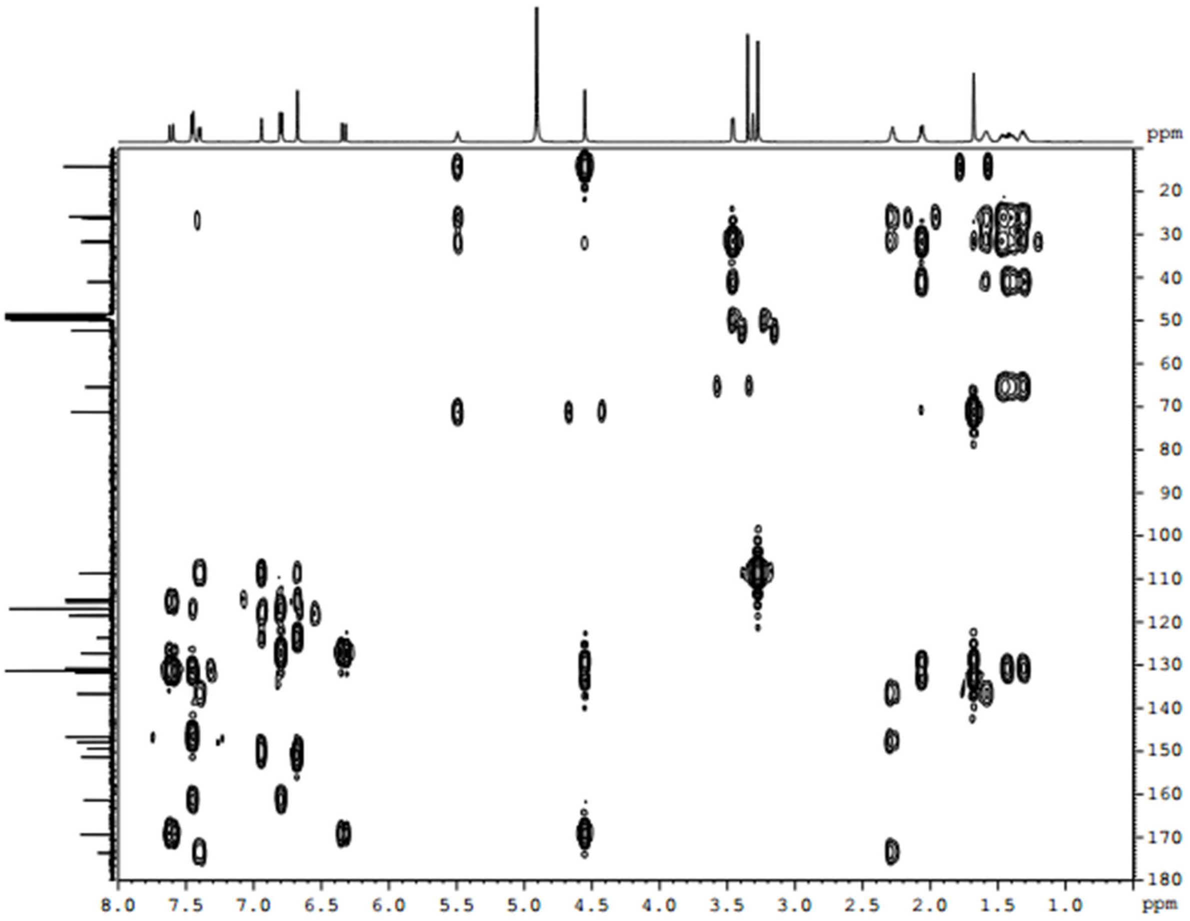

Supplementary Figure 4: HMBC spectrum of 1 and 2 in CD_3OD.

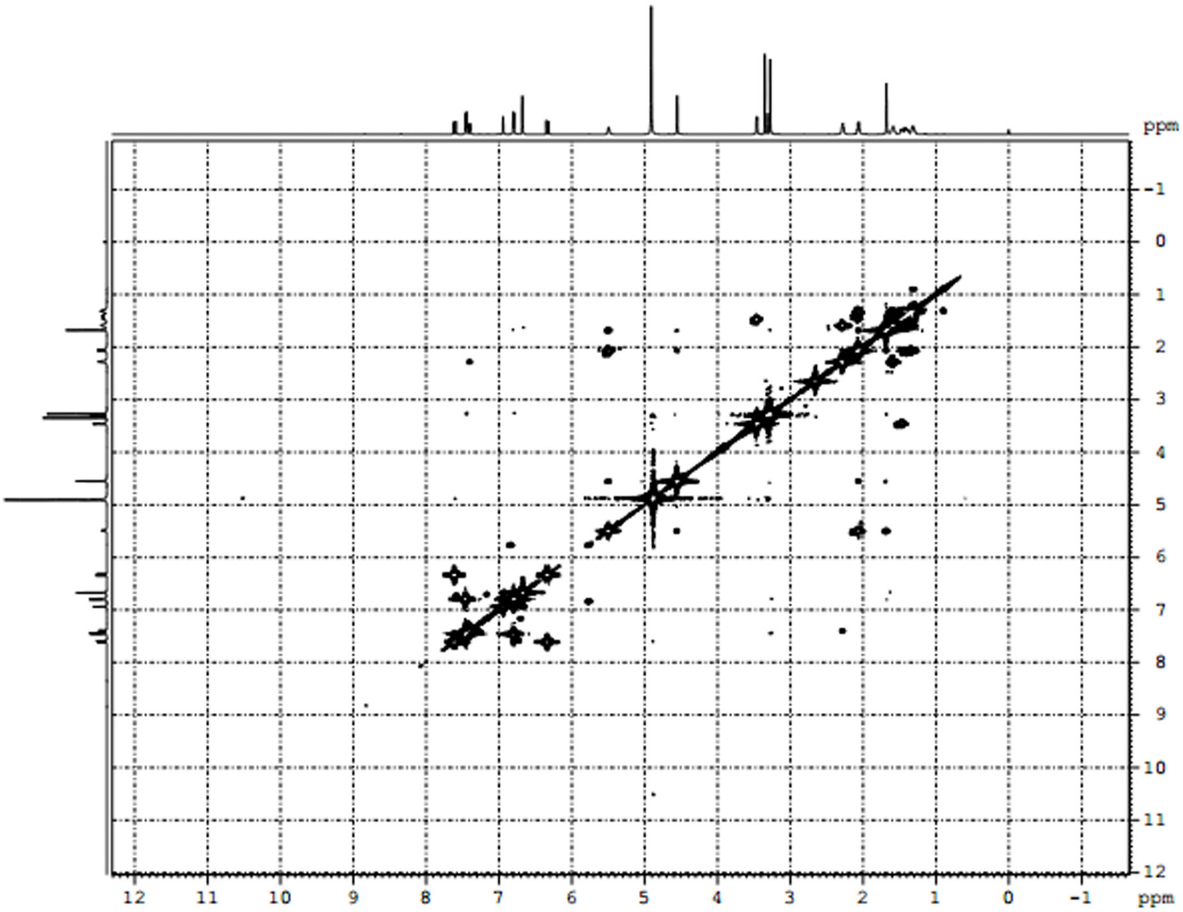

Supplementary Figure 5: ^{1}H-1H COSY spectrum of 1 and 2 in CD_3OD.

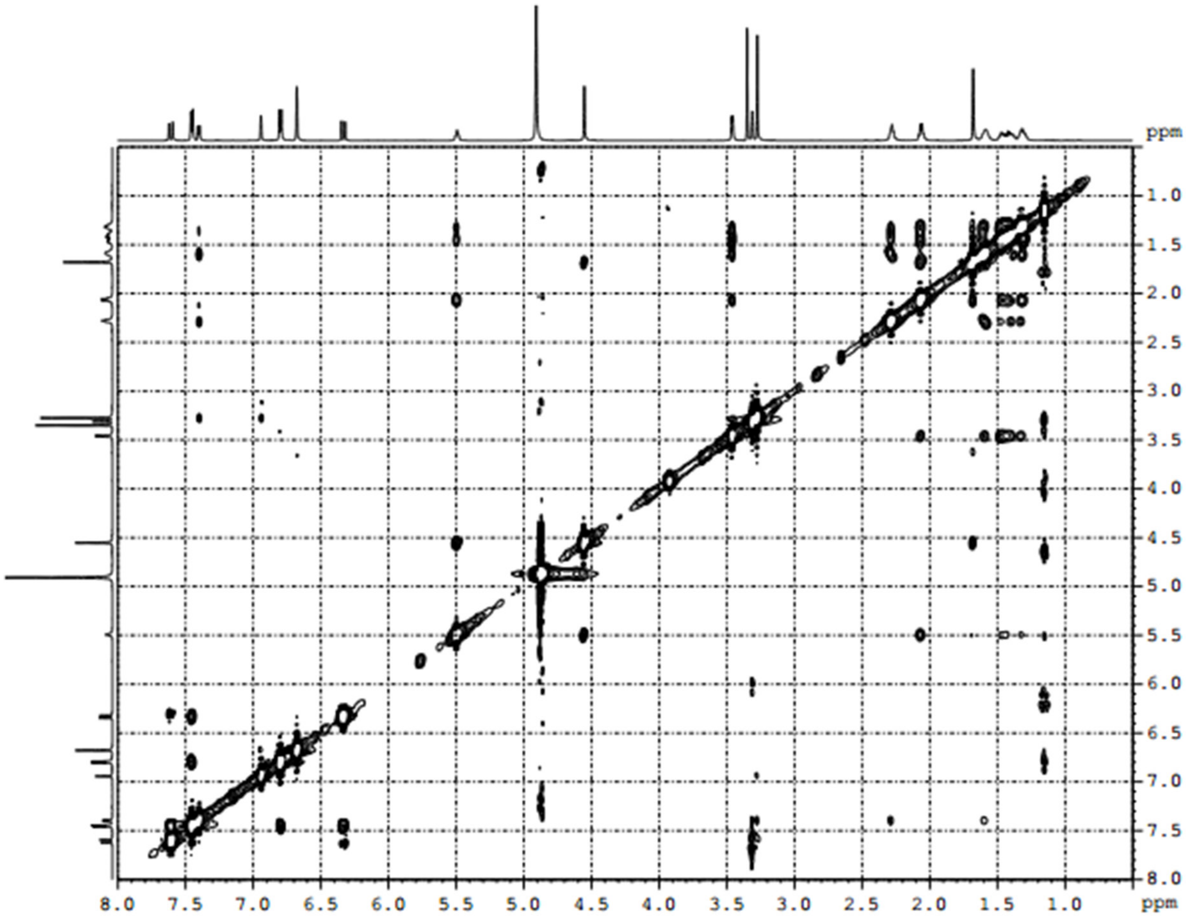

Supplementary Figure 6: NOESY spectrum of 1 and 2 in CD_3OD.

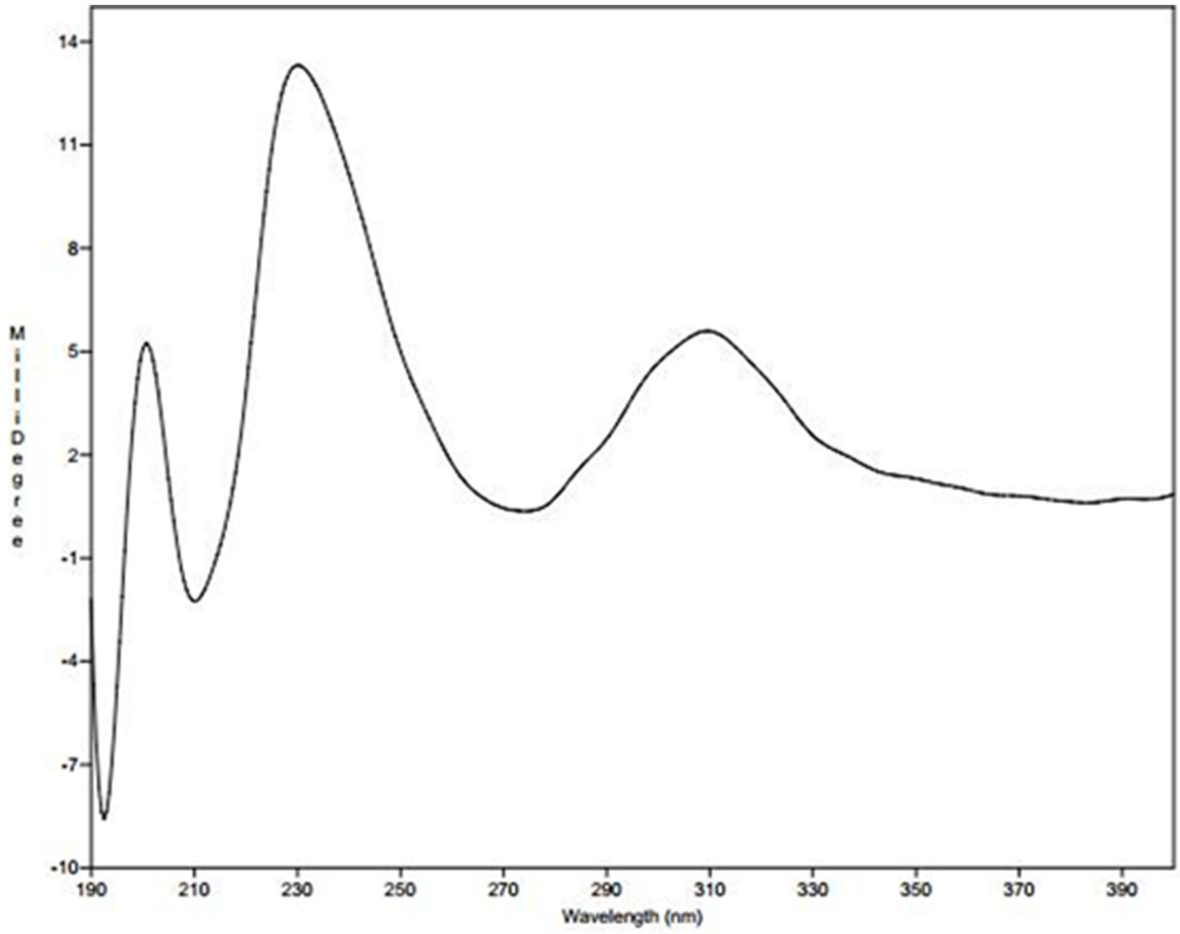

Supplementary Figure 7: CD spectrum of compound 1.

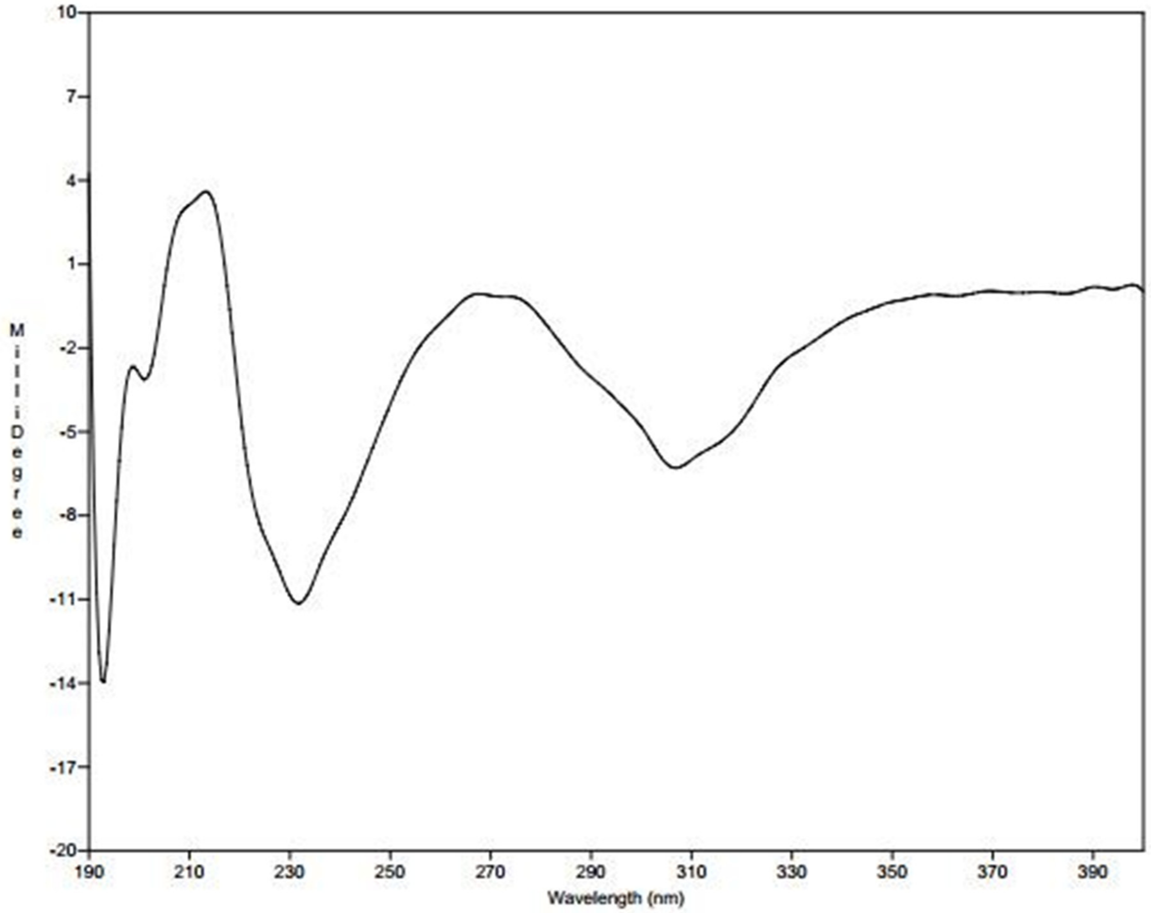

Supplementary Figure 8: CD spectrum of compound 2.

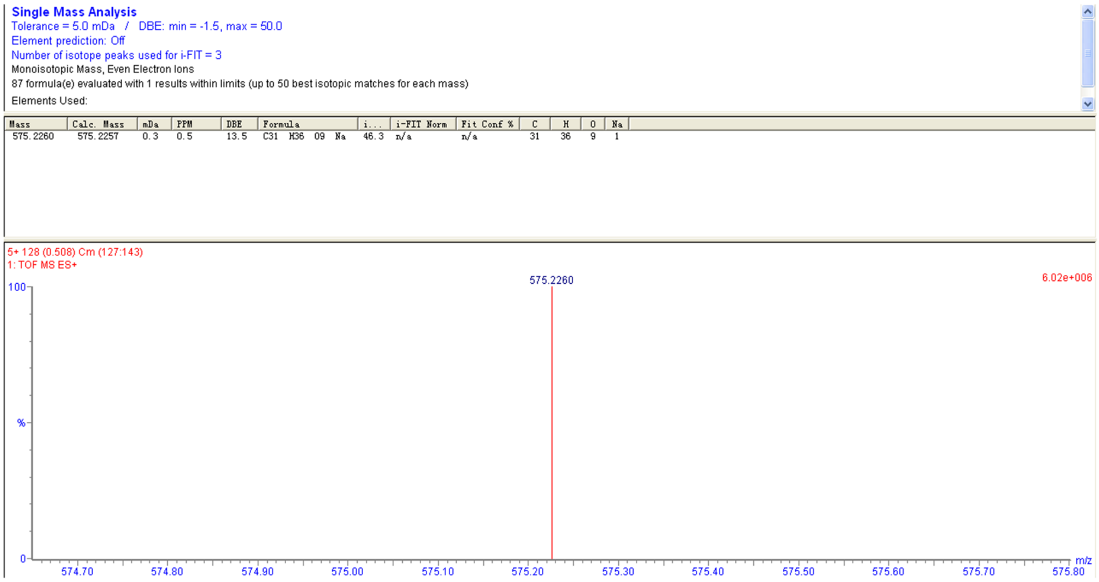

Supplementary Figure 9: HRESIMS data of 1 and 2.

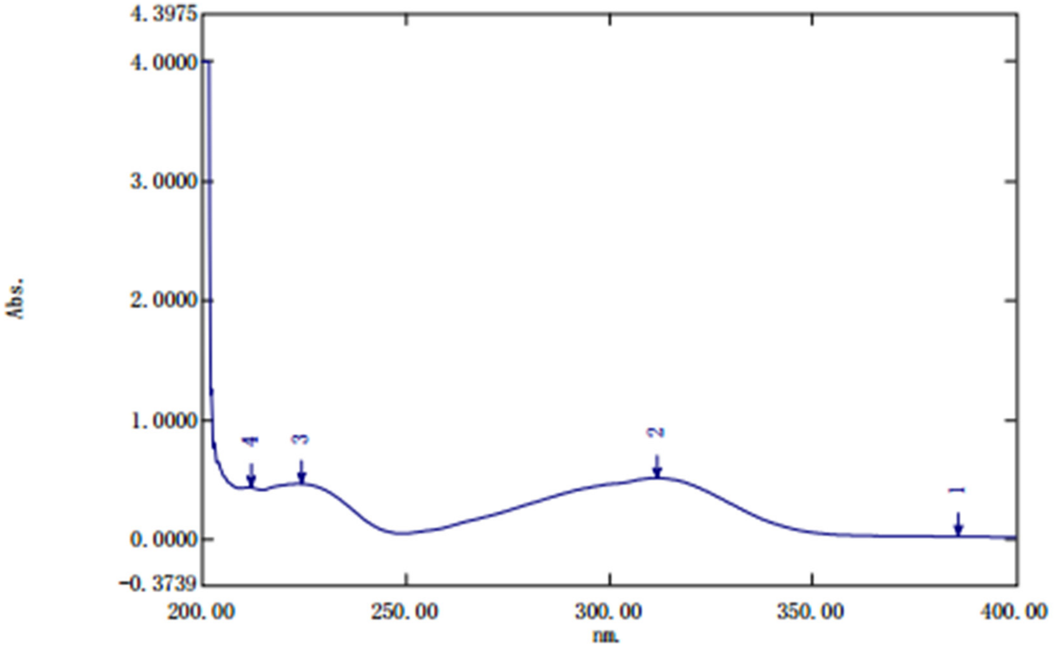

测定属性
波长范围（nm.）:　200.00到400.00
扫描速度:　中速
采样间隔:　0.2
自动采样间隔: 启用
扫描模式:　自动

试样准备属性
重量:
体积 :

No.	P/V	Wavelength	Abs.	描述
1	↑	386.00	.0292	
2	↑	311.60	.5199	
3	↑	224.20	.4701	
4	↑	211.80	.4410	
5	↓	361.60	.0364	
6	↓	248.40	.0542	
7	↓	214.20	.4185	
8	↓	210.00	.4332	

Supplementary Figure 10: UV spectrum of 1 and 2.

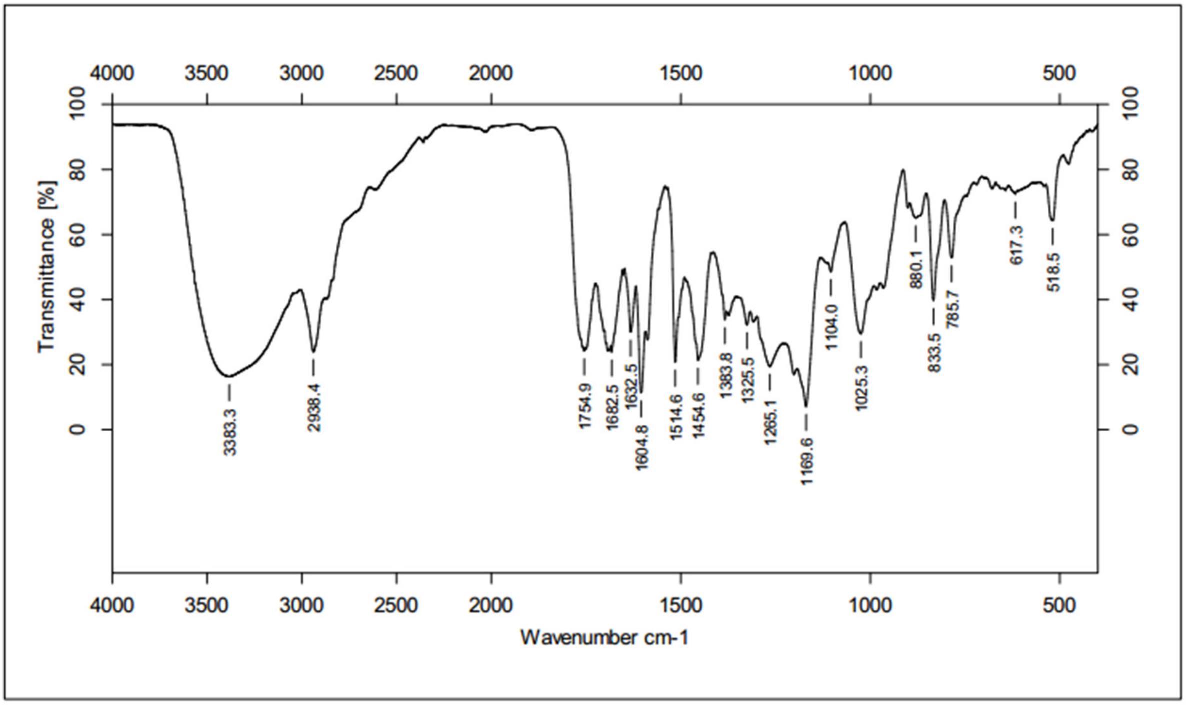

Sample : WM-E53342	Frequency Range : 3999.64 - 400.157	Measured on : 2015-3-24	
Technique : Sample form	Resolution : 2	Instrument : EQUINOX55	Sample Scans : 16
Customer : Default	Zerofilling : 2	Acquisition : Double Sided,Forv	

Supplementary Figure 11: IR spectrum of 1 and 2.

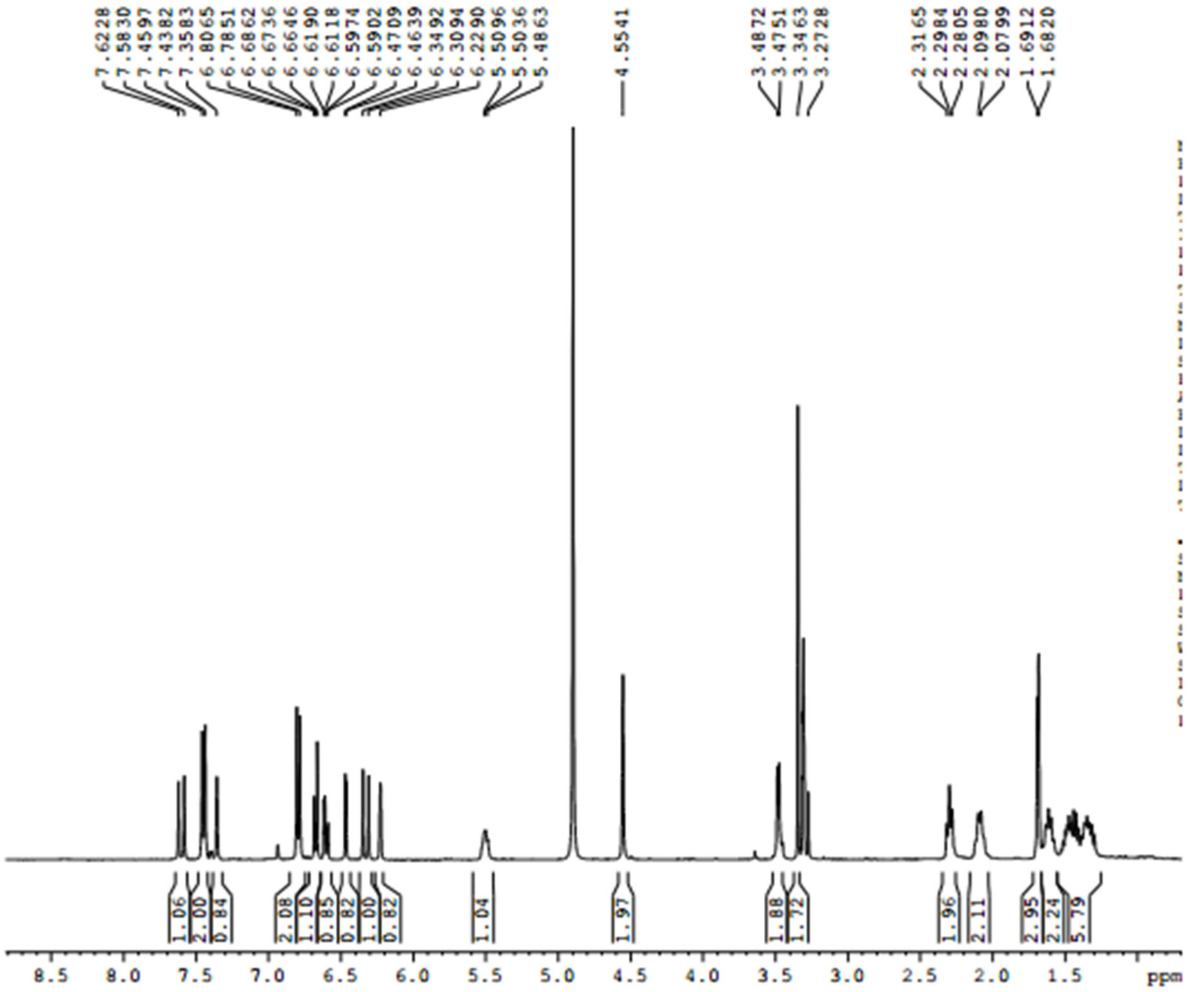

Supplementary Figure 12: ^{1}H NMR (400 MHz) spectrum of 3 and 4 in CD_3OD.

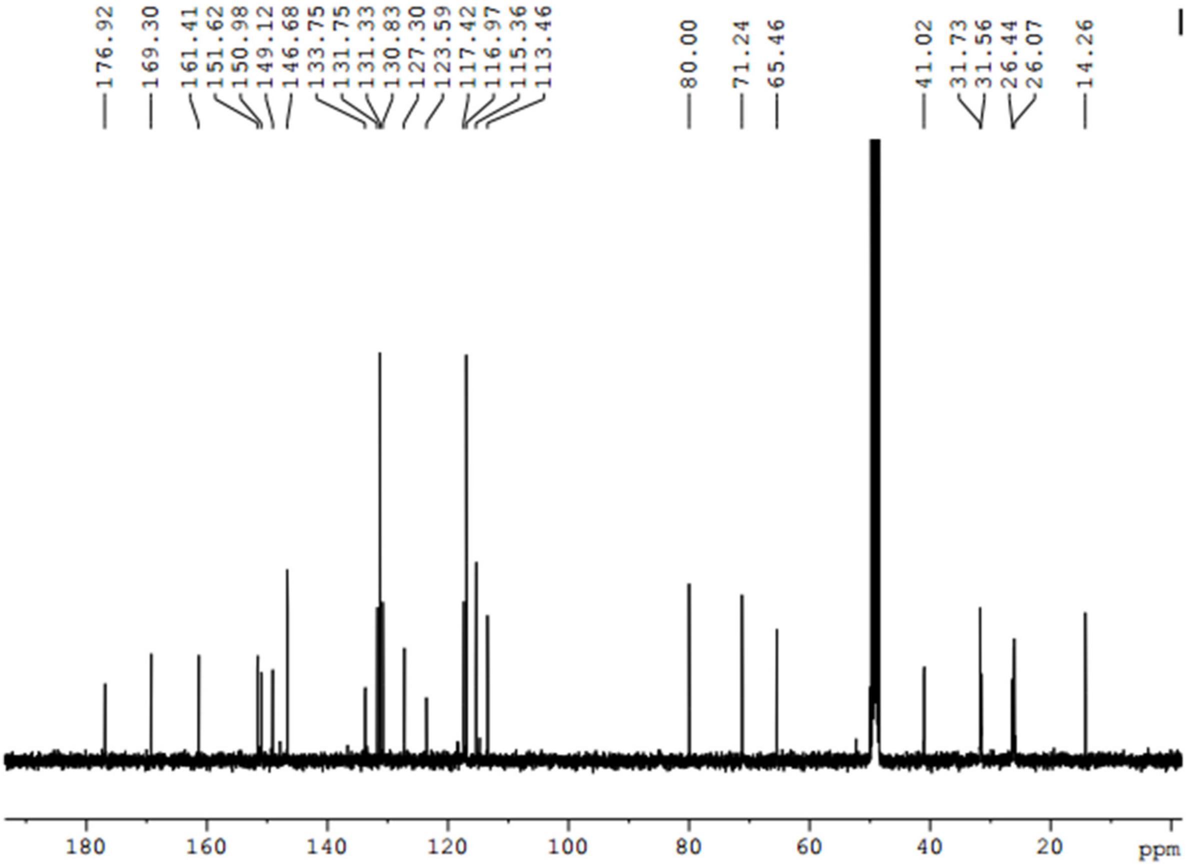

Supplementary Figure 13: ^{13}**C NMR (100 MHz) spectrum of 3 and 4 in** CD_3OD**.**

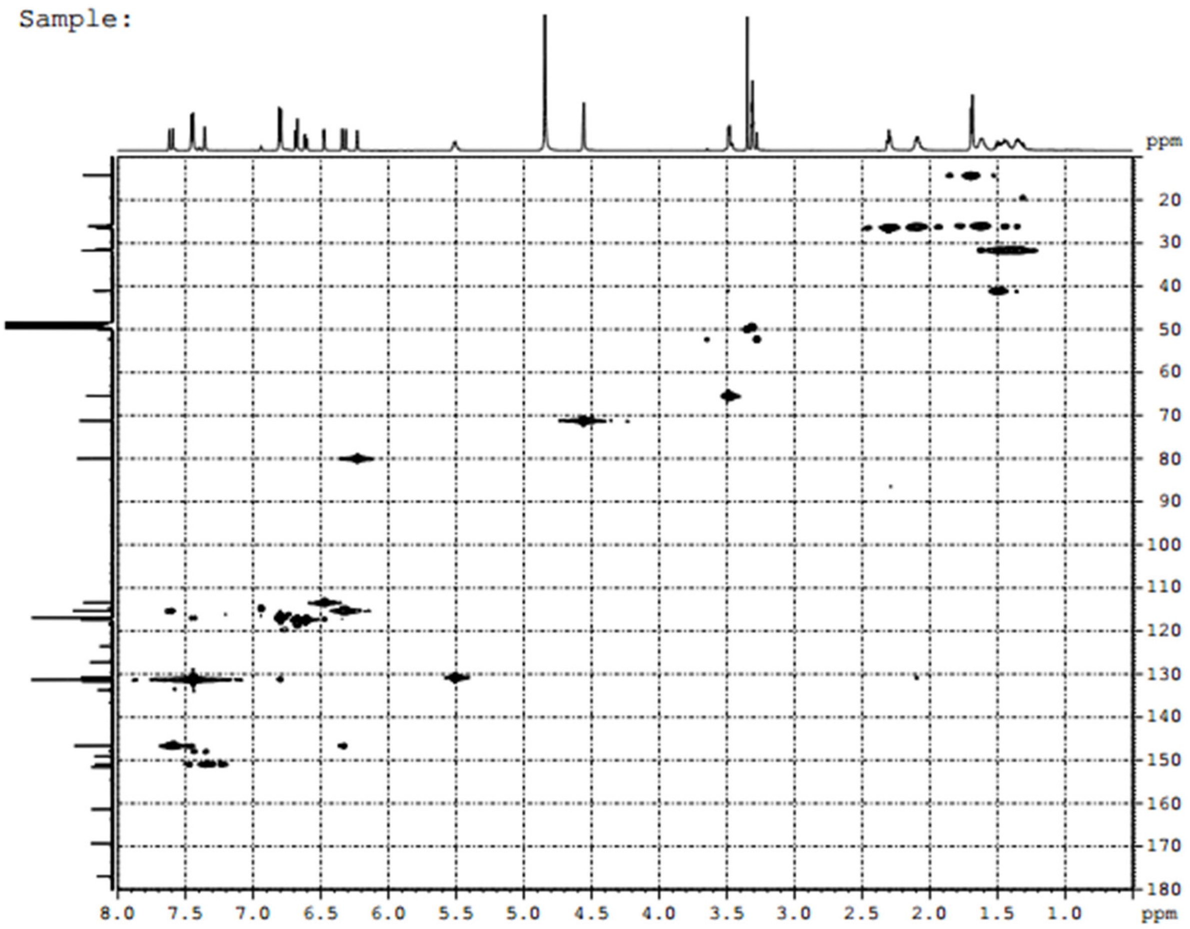

Supplementary Figure 14: HSQC spectrum of 3 and 4 in CD_3OD.

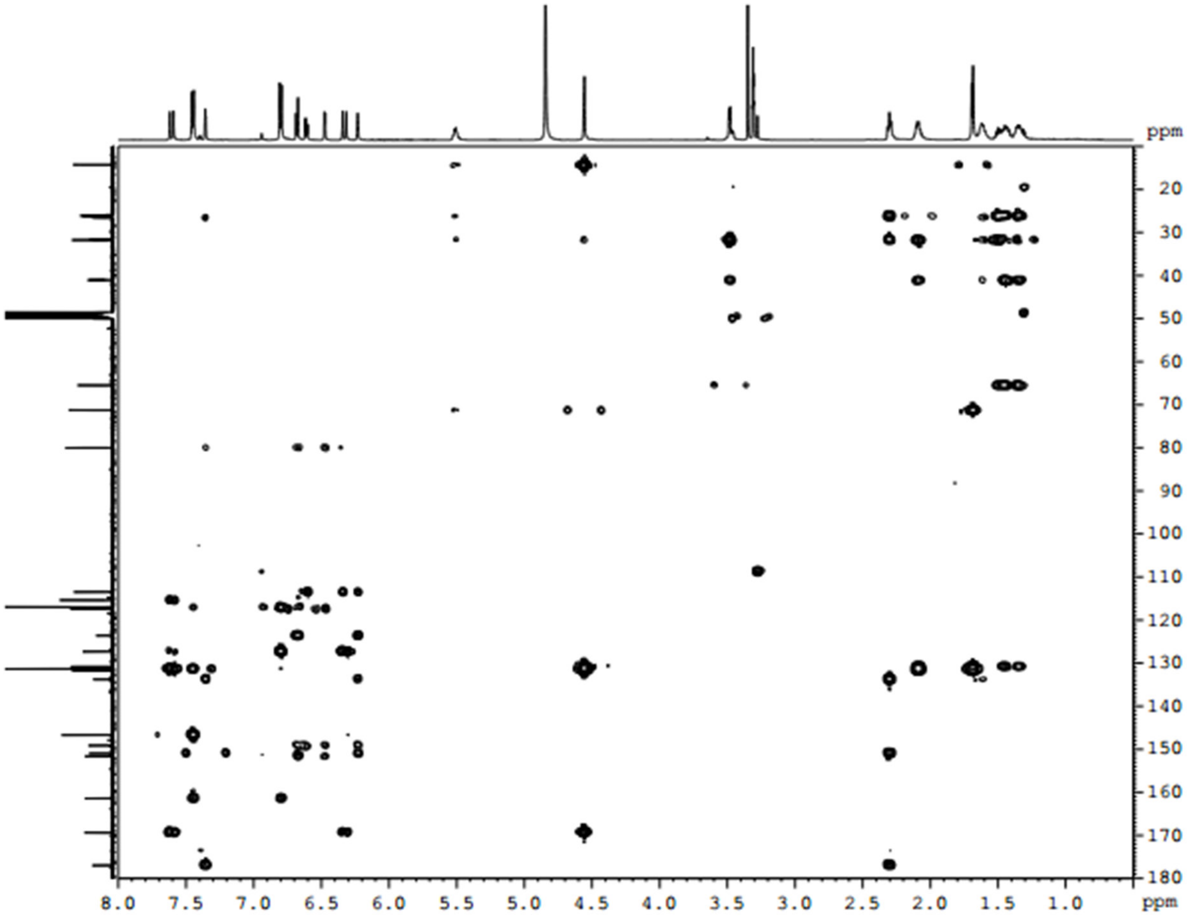

Supplementary Figure 15: HMBC spectrum of 3 and 4 in CD_3OD.

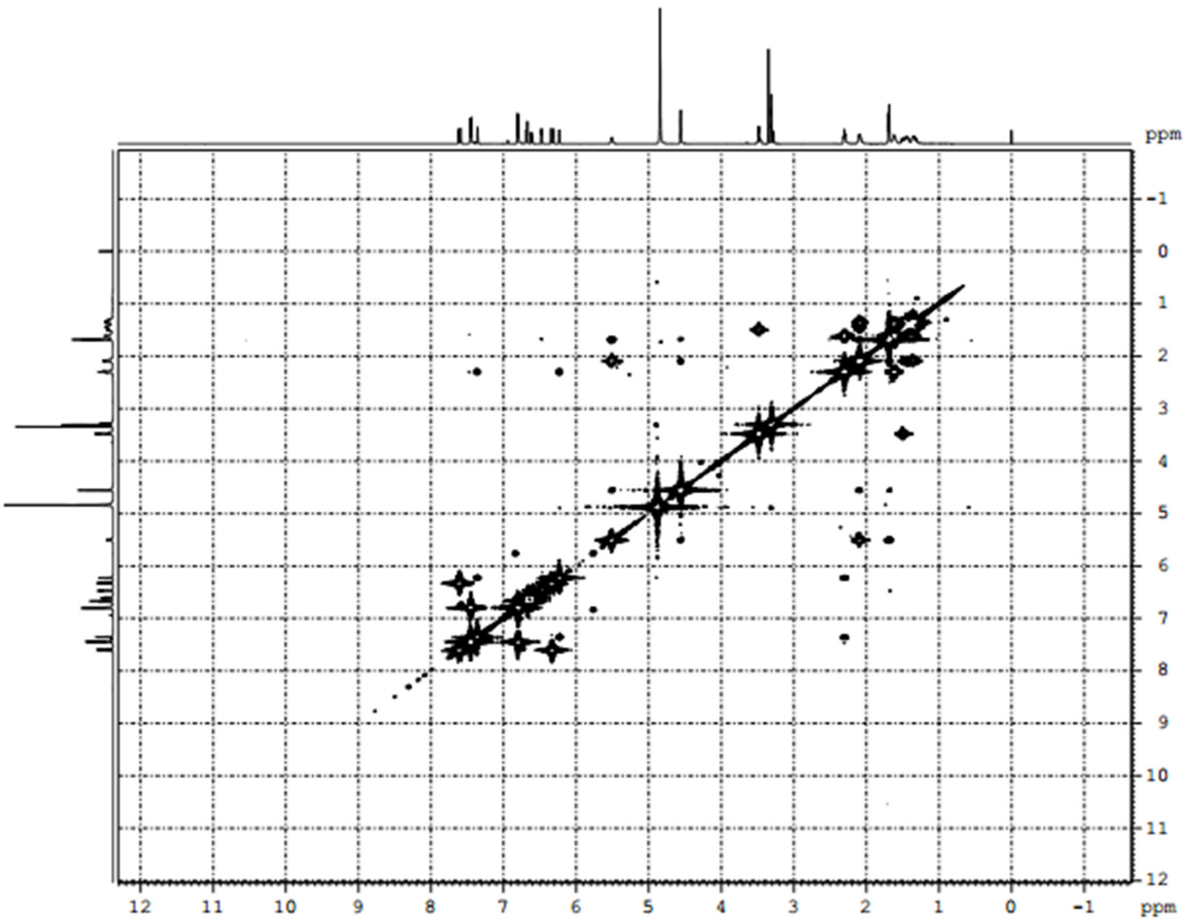

Supplementary Figure 16: ^{1}H-^{1}H COSY spectrum of 3 and 4 in CD_3OD.

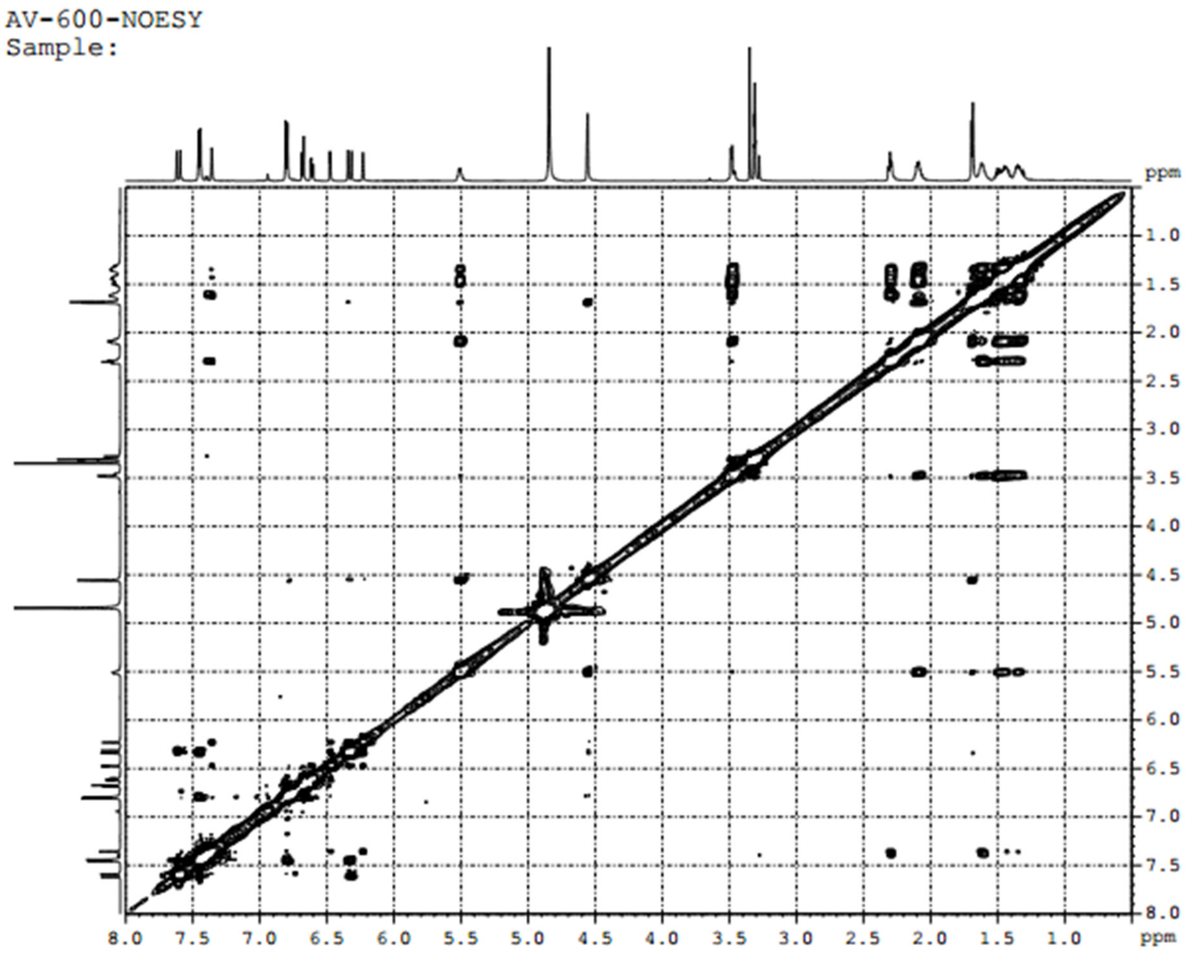

Supplementary Figure 17: NOESY spectrum of 3 and 4 in CD_3OD.

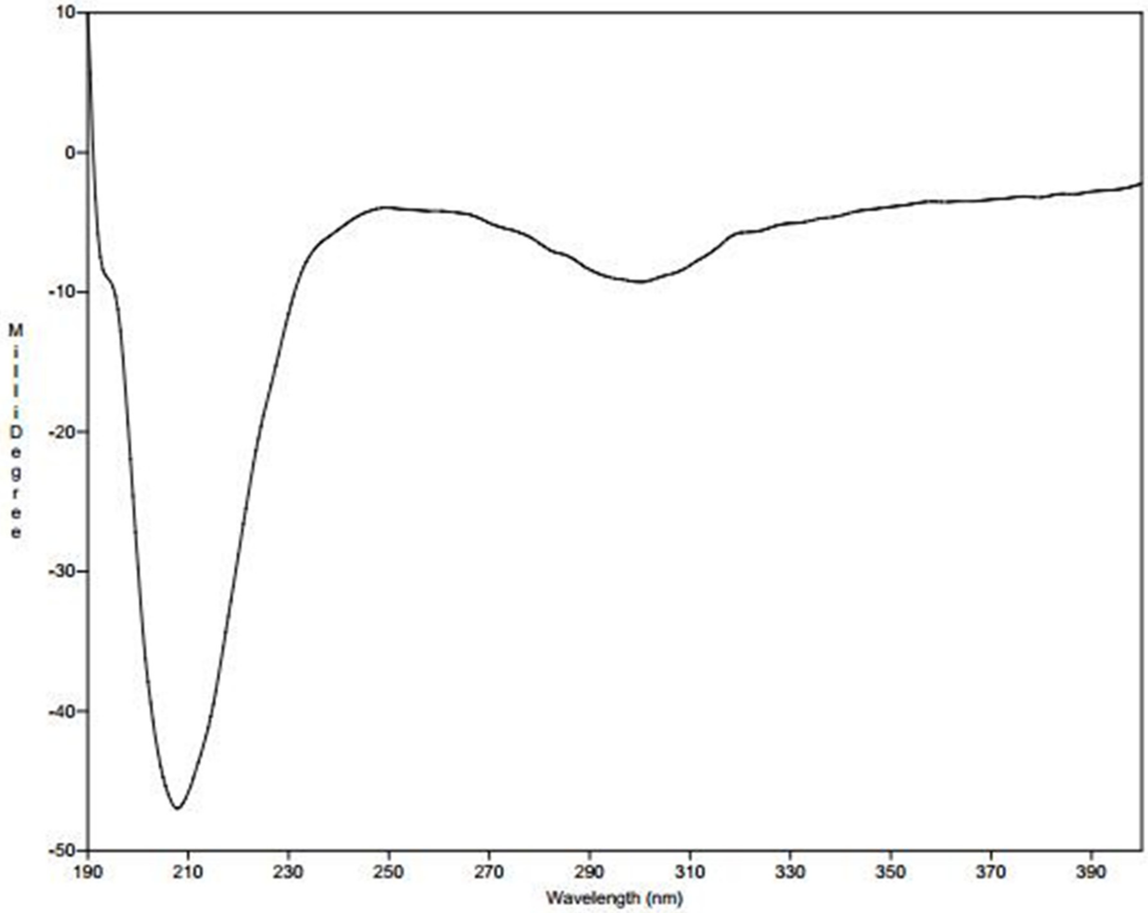

Supplementary Figure 18: CD spectrum of compound 3.

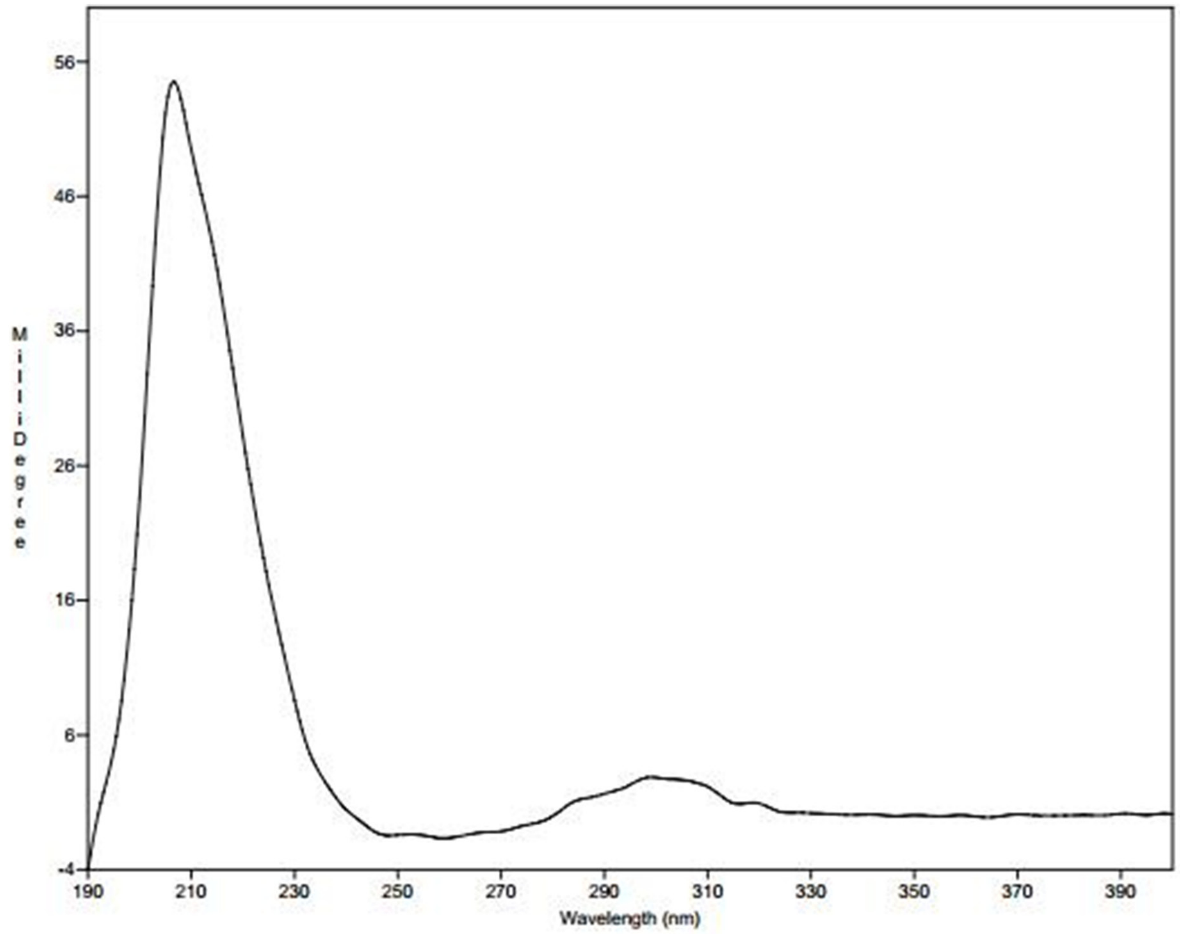

Supplementary Figure 19: CD spectrum of compound 4.

Single Mass Analysis
Tolerance = 5.0 mDa / DBE: min = -1.5, max = 50.0
Element prediction: Off
Number of isotope peaks used for i-FIT = 3
Monoisotopic Mass, Even Electron Ions
75 formula(e) evaluated with 1 results within limits (up to 50 best isotopic matches for each mass)
Elements Used:

Mass	Calc. Mass	mDa	PPM	DBE	Formula	i-FIT	i-FIT Norm	Fit Conf %	C	H	O	Na
545.2153	545.2151	0.2	0.4	13.5	C30 H34 O8 Na	110.7	n/a	n/a	30	34	8	1

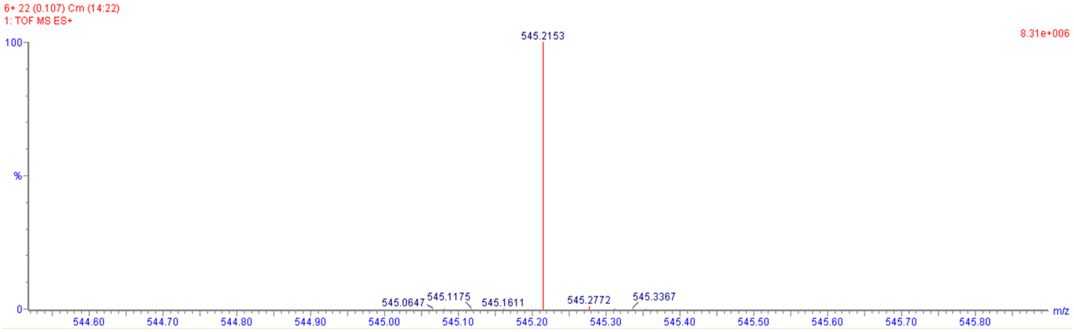

Supplementary Figure 20: HRESIMS data of 3 and 4.

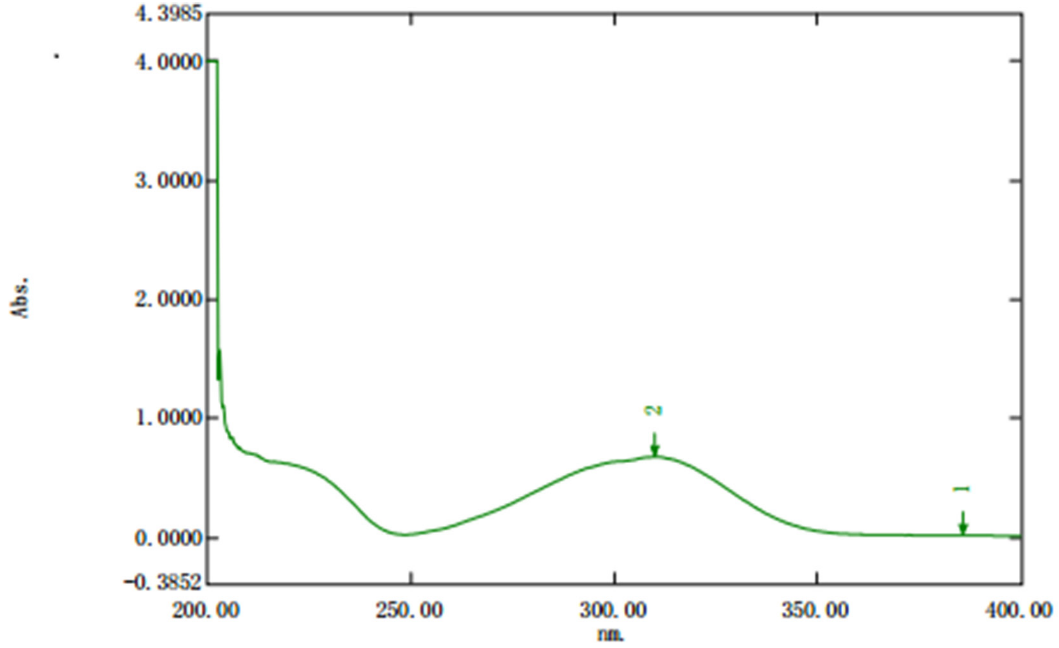

测定属性
波长范围 (nm.): 200.00到400.00
扫描速度: 中速
采样间隔: 0.2
自动采样间隔: 启用
扫描模式: 自动

No.	P/V	Wavelength	Abs.	描述
1	↑	386.00	.0177	
2	↑	310.00	.6787	
3	↓	371.80	.0193	
4	↓	248.60	.0234	

Supplementary Figure 21: UV spectrum of 3 and 4.

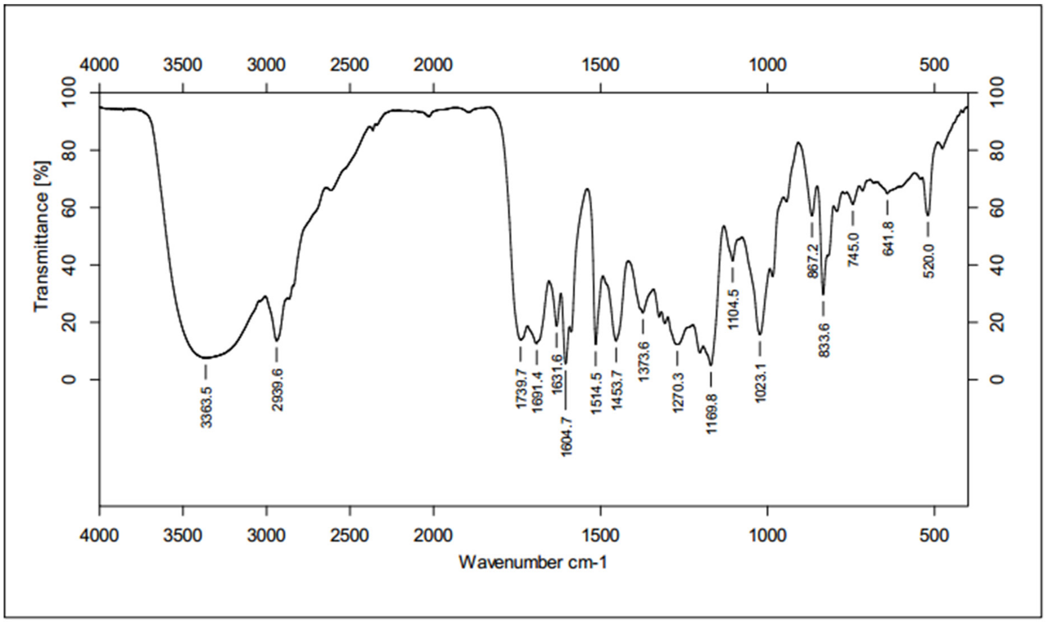

Sample : WM-E53442	Frequency Range : 3999.64 - 400.157	Measured on : 2015-3-24	
Technique : Sample form	Resolution : 2	Instrument : EQUINOX55	Sample Scans : 16
Customer : Default	Zerofilling : 2	Acquisition : Double Sided,Forv	

Supplementary Figure 22: IR spectrum of 3 and 4.

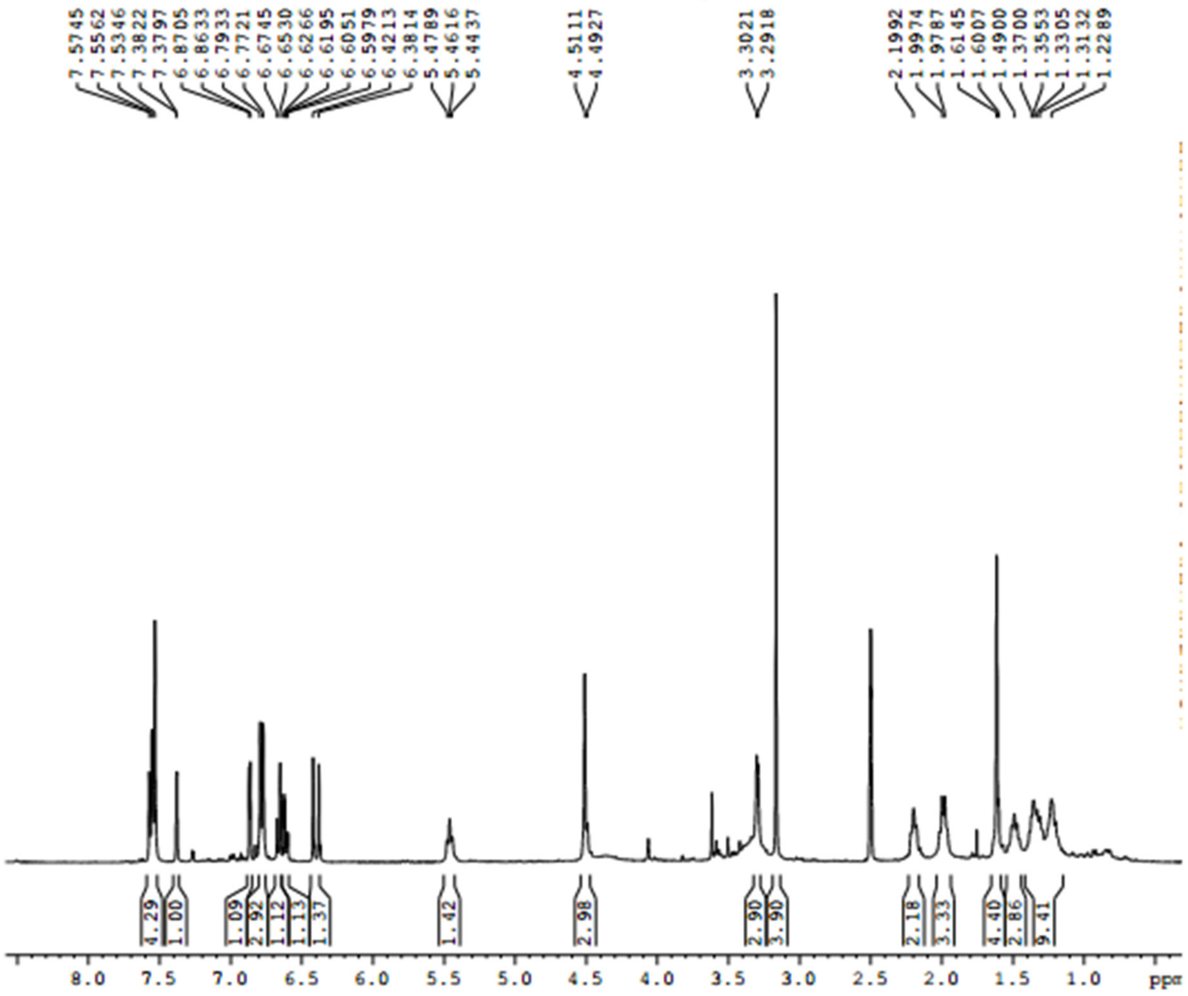

Supplementary Figure 23: ^{1}H NMR (400 MHz) spectrum of 5 and 6 in DMSO-d_6.

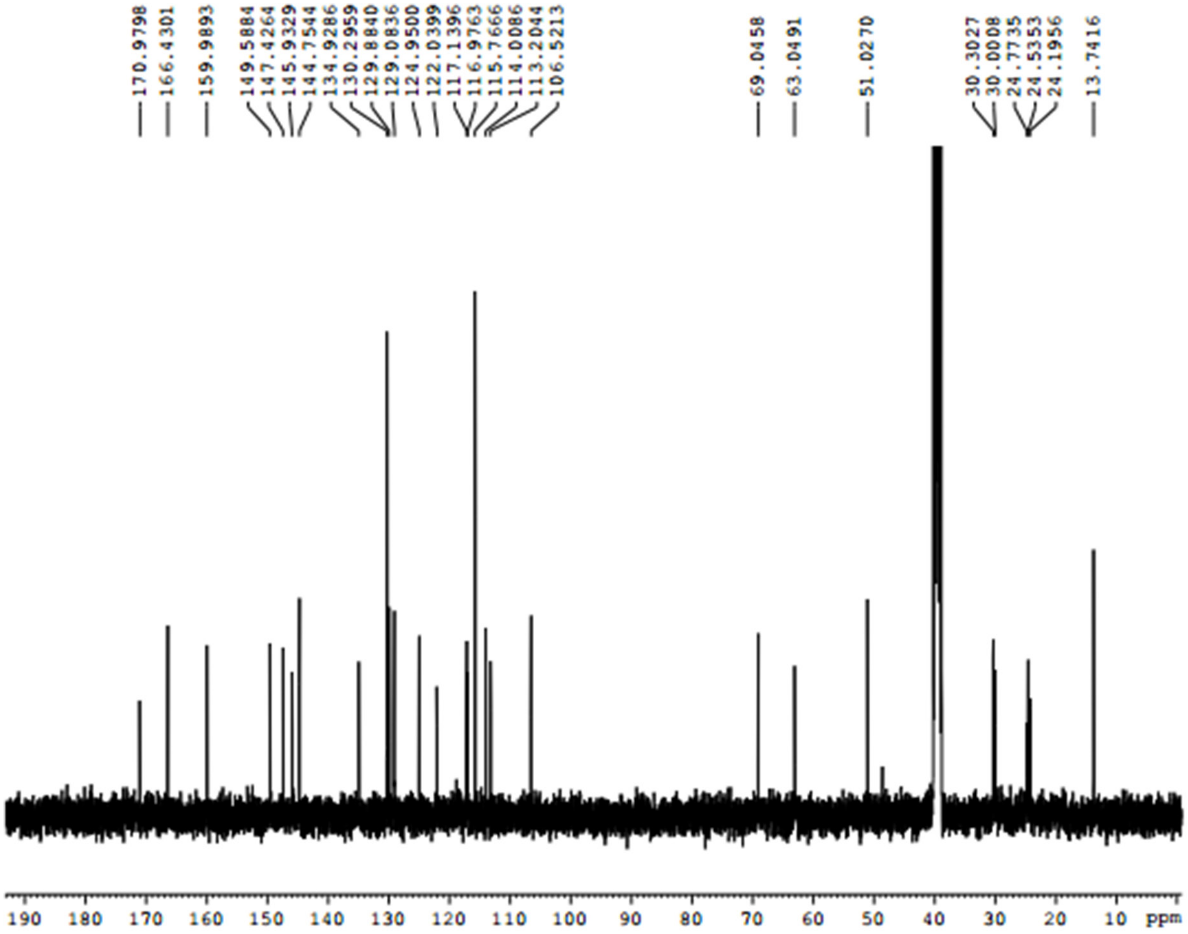

Supplementary Figure 24: ^{13}**C NMR (100 MHz) spectrum of 5 and 6 in DMSO-**d_6**.**

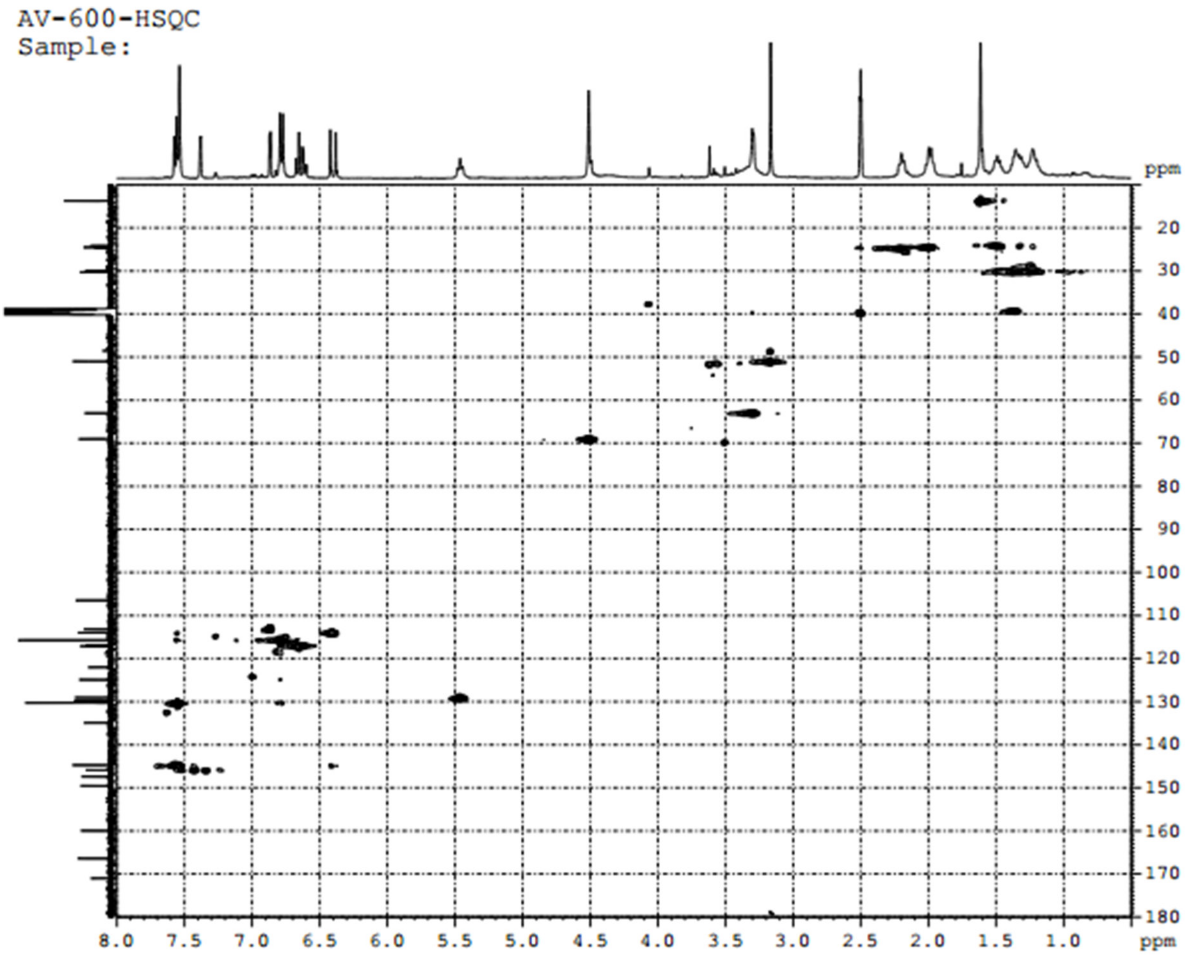

Supplementary Figure 25: HSQC spectrum of 5 and 6 in DMSO-d_6.

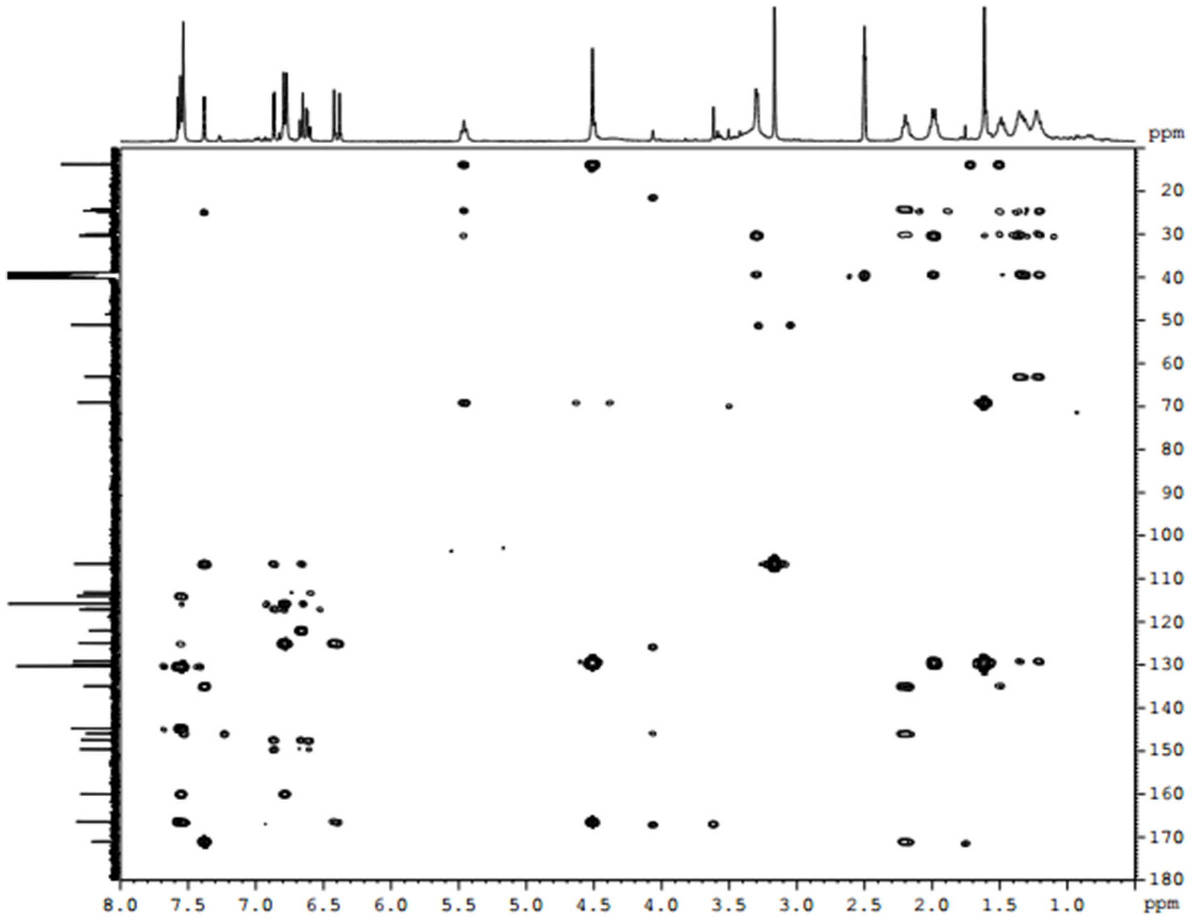

Supplementary Figure 26: HMBC spectrum of 5 and 6 in DMSO-d_6.

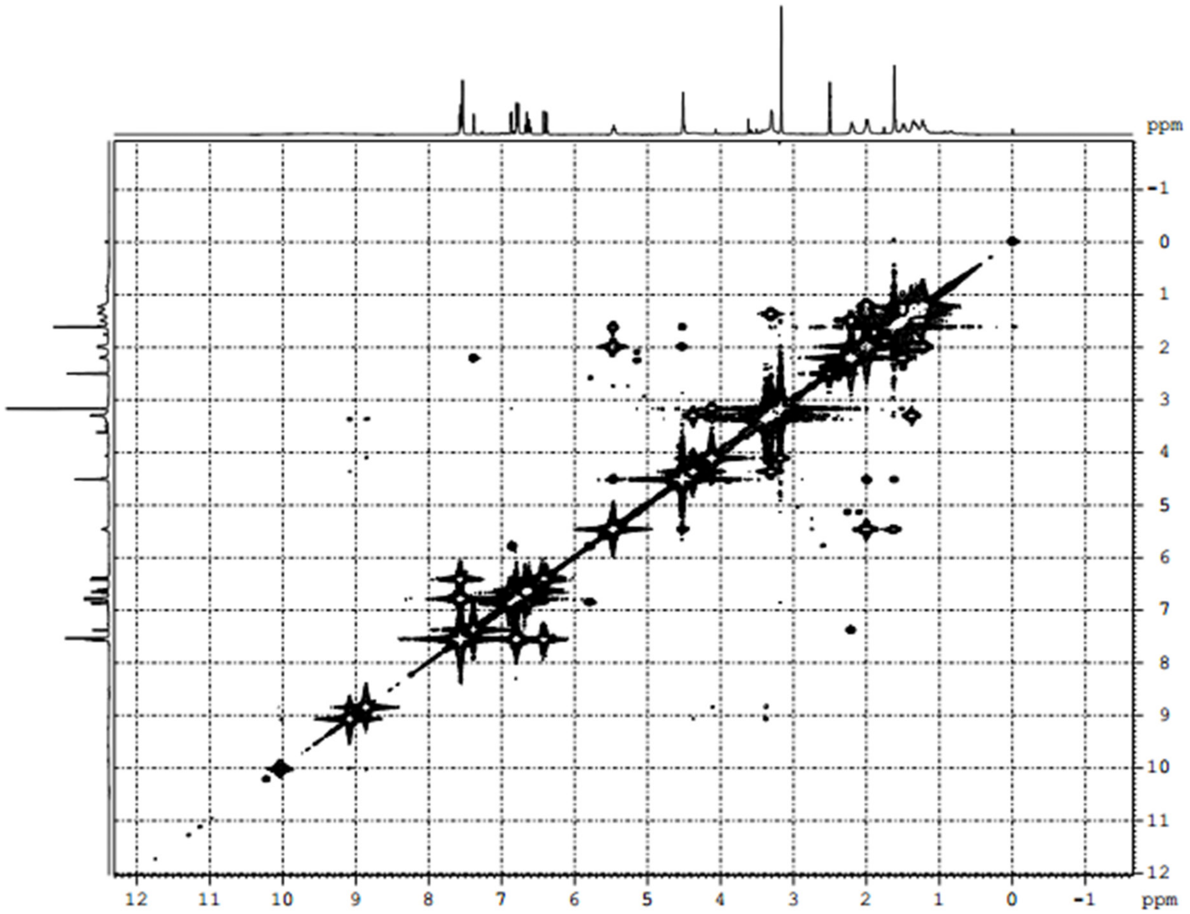

Supplementary Figure 27: ^{1}H-^{1}H COSY spectrum of 5 and 6 in DMSO-d_6.

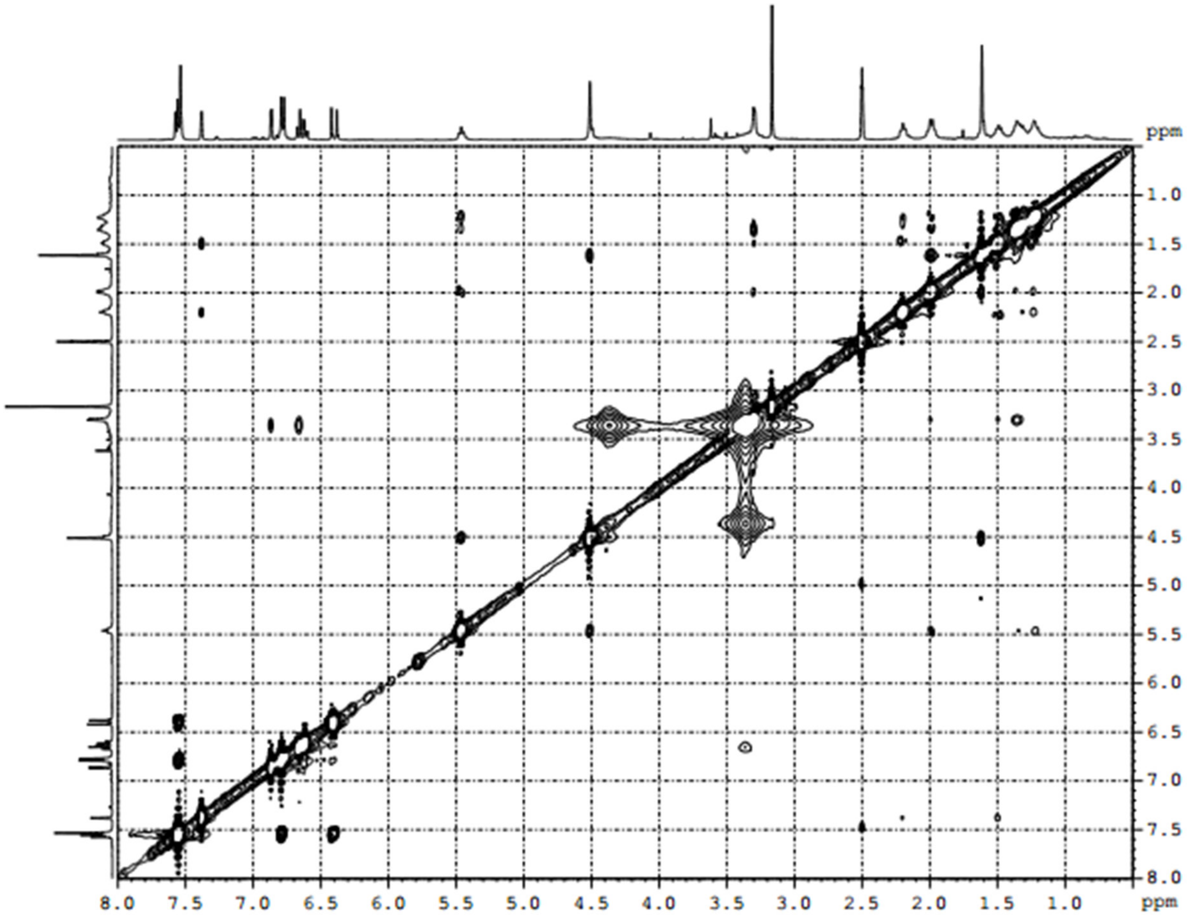

Supplementary Figure 28: NOESY spectrum of 5 and 6 in DMSO-d_6.

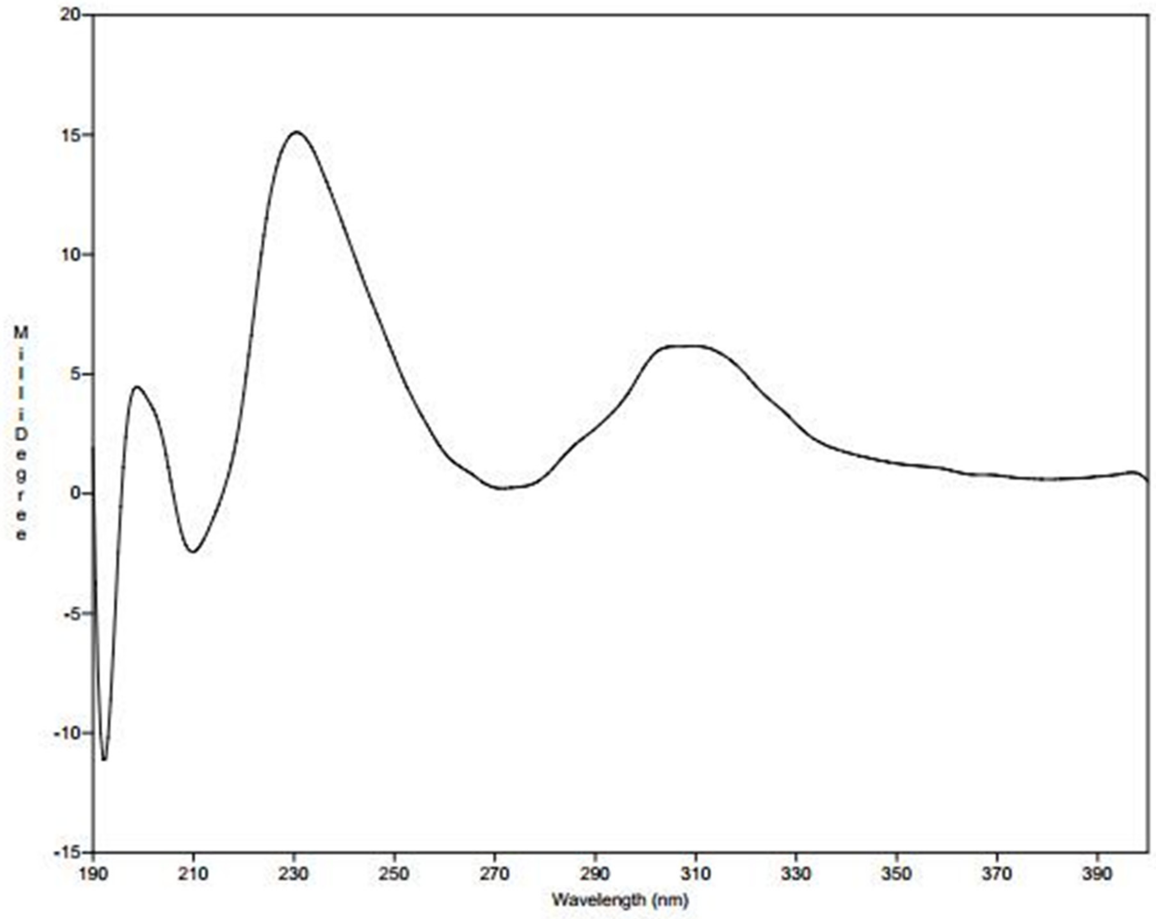

Supplementary Figure 29: CD spectrum of compound 5.

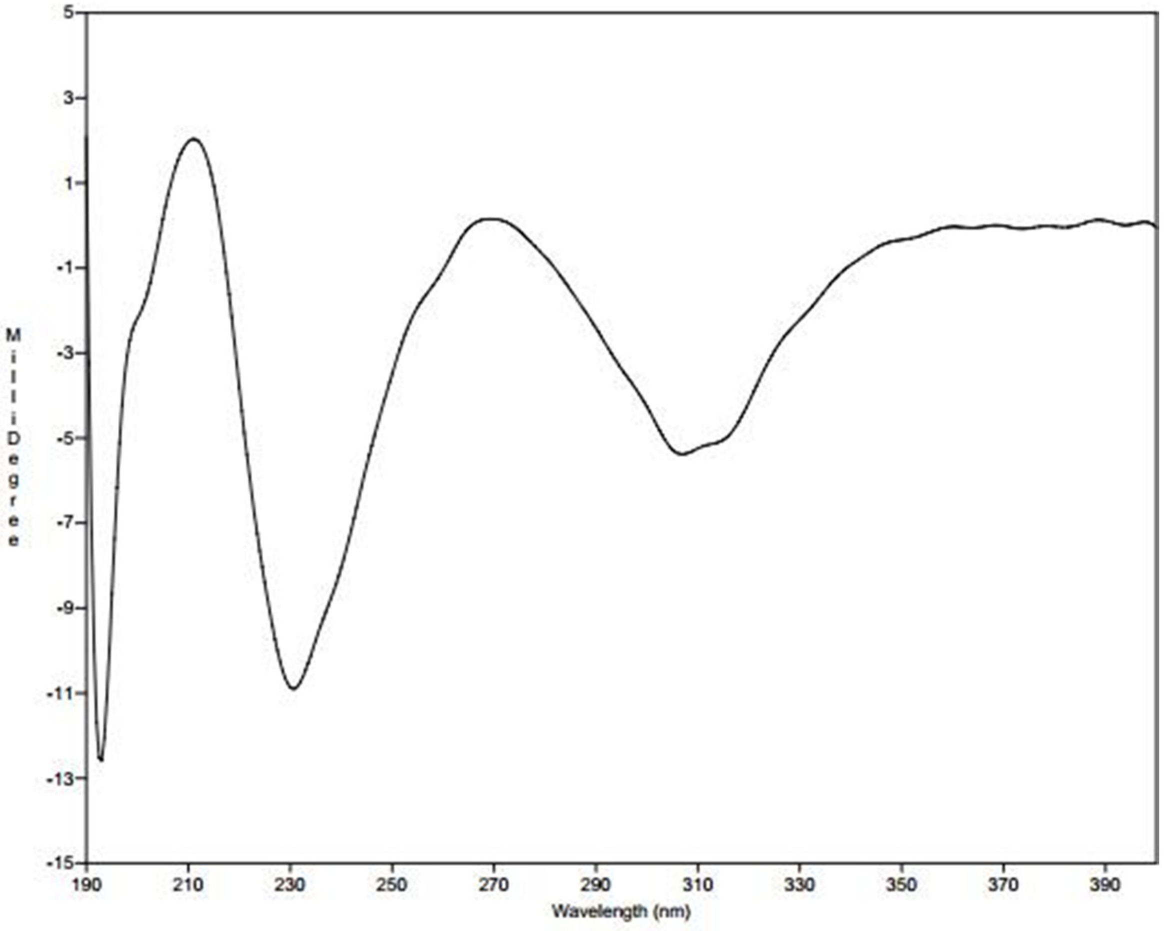

Supplementary Figure 30: CD spectrum of compound 6.

Single Mass Analysis
Tolerance = 5.0 mDa / DBE: min = -1.5, max = 50.0
Element prediction: Off
Number of isotope peaks used for i-FIT = 3
Monoisotopic Mass, Even Electron Ions
79 formula(e) evaluated with 1 results within limits (up to 50 best isotopic matches for each mass)
Elements Used:

Mass	Calc. Mass	mDa	PPM	DBE	Formula	i-FIT	i-FIT Norm	Fit Conf %	C	H	O	Na
575.2261	575.2257	0.4	0.7	13.5	C31 H36 O9 Na	281.0	n/a	n/a	31	36	9	1

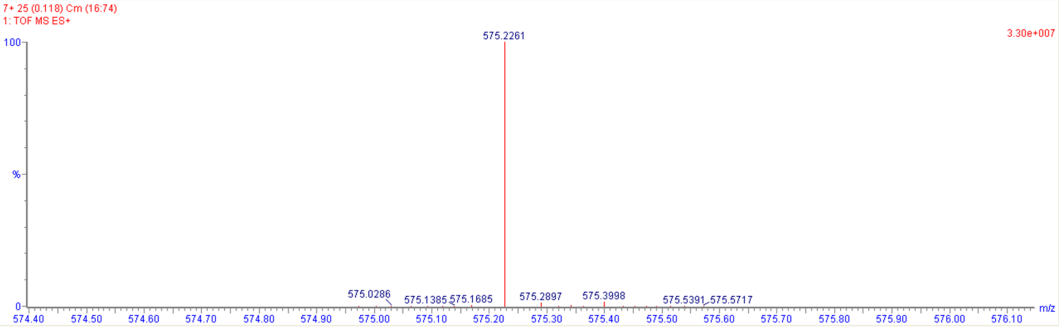

Supplementary Figure 31: HRESIMS data of 5 and 6.

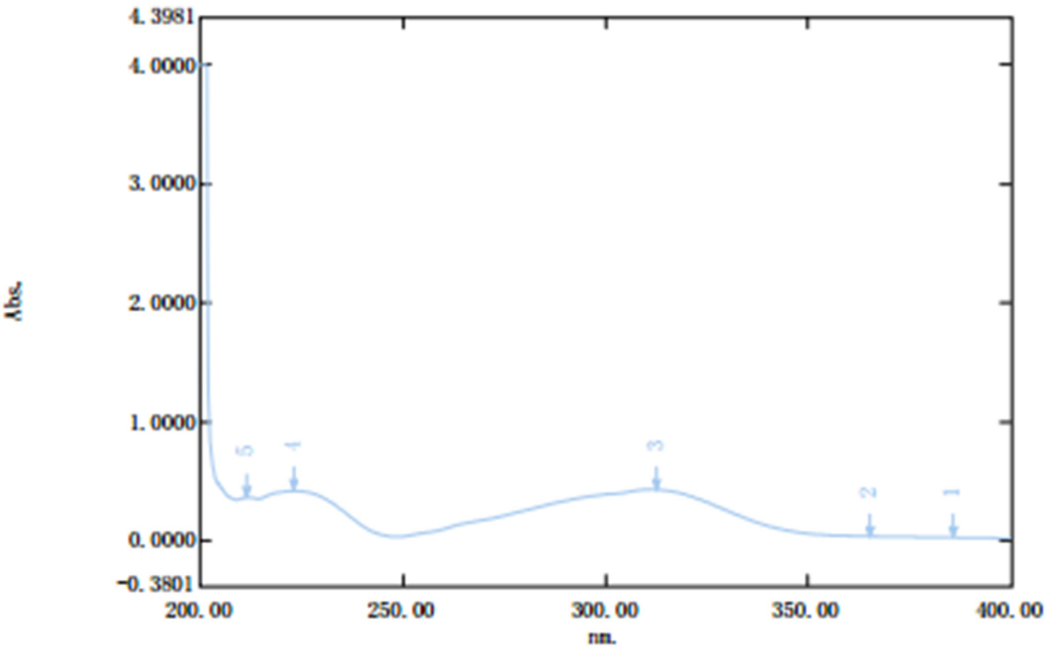

测定属性
波长范围 (nm.): 200.00到400.00
扫描速度: 中速
采样间隔: 0.2
自动采样间隔: 启用
扫描模式: 自动

试样准备属性
重量:
体积 :
稀释:
光程长:
附加信息:

No.	P/V	Wavelength	Abs.	描述
1		386.00	.0255	
2		365.40	.0348	
3		312.60	.4294	
4		223.20	.4175	
5		211.40	.3634	
6		364.60	.0342	
7		248.20	.0298	
8		214.20	.3484	
9		208.80	.3409	

Supplementary Figure 32: UV spectrum of 5 and 6.

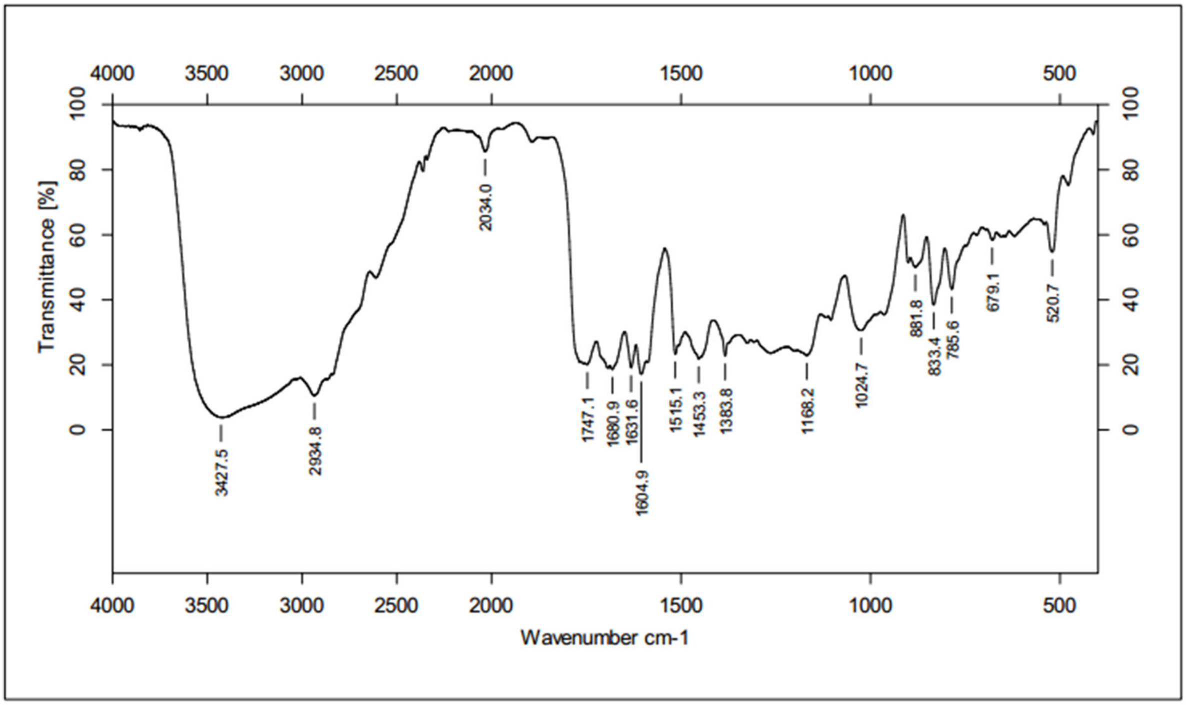

Sample : WM-E53444	Frequency Range : 3999.64 - 400.157	Measured on : 2015-3-24	
Technique : Sample form	Resolution : 2	Instrument : EQUINOX55	Sample Scans : 16
Customer : Default	Zerofilling : 2	Acquisition : Double Sided,Forv	

Supplementary Figure 33: IR spectrum of 5 and 6.
